# Nitrogen Enriched Tröger’s Base Polymers of Intrinsic Microporosity for Heterogeneous Catalysis

**DOI:** 10.1021/acsapm.4c02952

**Published:** 2024-12-18

**Authors:** Natasha Hawkins, Ariana R. Antonangelo, Mitchell Wood, Elena Tocci, Johannes Carolus Jansen, Alessio Fuoco, Carmen Rizzuto, Mariagiulia Longo, C. Grazia Bezzu, Mariolino Carta

**Affiliations:** † Department of Chemistry, Faculty of Science and Engineering, Swansea University, Grove Building, Singleton Park, Swansea SA2 8PP, U.K.; ‡ Institute on Membrane Technology, National Research Council of Italy (CNR-ITM), via P. Bucci 17/C, Rende (CS) 87036, Italy

**Keywords:** heterogeneous catalysis, Tröger’s base, PIMs, Knoevenagel, microporosity

## Abstract

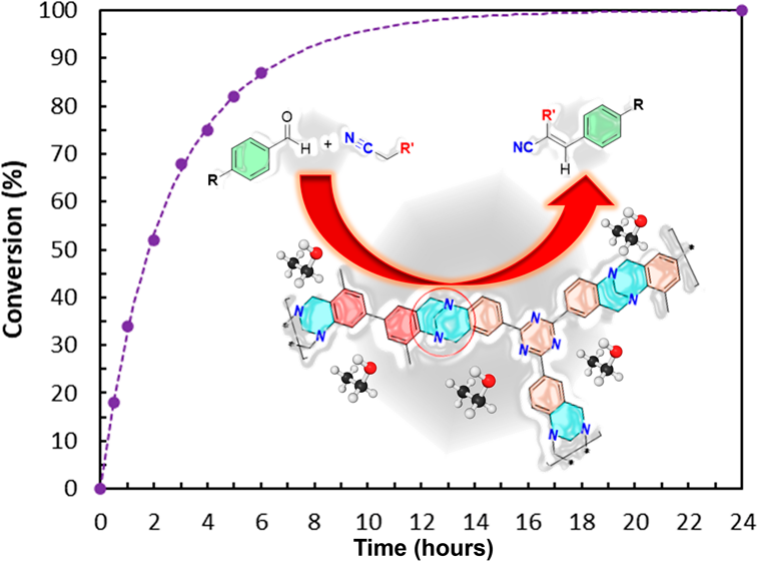

Heterogeneous catalysis is significantly enhanced by the use of highly porous polymers with specific functionalities, such as basic groups, which accelerate reaction rates. Polymers of intrinsic microporosity (PIMs) provide a unique platform for catalytic reactions owing to their high surface areas and customizable pore structures. We herein report a series of Tröger’s base polymers (TB-PIMs) with enhanced basicity, achieved through the incorporation of nitrogen-containing groups into their repeat units, such as triazine and triphenylamine. These polymers offer a perfect balance between the pore “swellability”, which allows the use of substrates of various dimensions, and the basicity of their repeat units, which facilitates the use of reactants with diverse acidity. The catalytic activity is evaluated through the Knoevenagel condensation of benzaldehydes and various methylene species, conducted in the presence of ethanol as a green solvent and using a 1:1 ratio of the two reagents. The results highlight a significant improvement, with reactions reaching completion using just a 1% molar ratio of catalysts and achieving a 3-fold enhancement over previous results with 4-*tert*-butyl-benzaldehyde. Computational modeling confirms that the enhanced basicity of the repeat units is attributable to the polymer design. Additionally, preliminary studies are undertaken to assess the kinetics of the catalyzed condensation reaction.

## 1 Introduction

In the quest for environmentally friendly and sustainable chemical processes, catalysis stands out as a foundation of progress as it not only enhances resource efficiency and reduces waste but also promotes selectivity and safety in chemical reactions, reducing energy consumption, cutting production costs, and minimizing environmental impact.^1,2^ Most importantly, catalysis embodies one of the pillars of the principles of green chemistry and sustainability.^3−5^ Nowadays, it plays a central role in a variety of industrial processes and laboratory experiments. Researchers consistently report the synthesis of new catalysts or the enhancement of the performance of existing ones, which are then applied to a wide range of commercially important reactions. Catalysis is also widely used in organic chemistry, both in academia and in industry, to improve the efficiency of the synthesis of added-value compounds. For example, developments were reported on the use of catalysts for very trending topics, such as the formation of new carbon–carbon bonds and C–H activation, which are growing fields of study both in the pharmaceutical industry and in general organic chemistry. In this context, Li et al. recently reported improved enantioselective Grubbs catalysts for asymmetric olefin metathesis,^6^ while Chao and co-workers increased the efficiency of C–H arylation of aldehydes containing aromatic moieties, exploiting Suzuki–Miyaura cross-coupling.^7^

The ongoing growth of the field translates into greener synthesis methods,^8^ eco-friendly materials,^9^ and a constantly evolving field that continues to lead in tackling the changing challenges of the modern world.^10^ This is particularly evident when considering it from the point of materials chemistry and polymer design, disciplines that are increasingly critical in a world acutely conscious of environmental challenges.^11^ In particular, polymer design is linked to the choice of heterogeneous catalysts over their homogeneous counterparts. The latter are often depicted as more active, but the big advantage of heterogeneous catalysis lies in their easier reusability and the more rapid separation and purification of the final products.^12^ Frequently efficient homogeneous catalysts are grafted onto preformed supports to overcome the problem of recyclability, but these systems are often affected by the leaching of the active site in solution and low thermal stability.^13^

Because of these advantages, heterogeneous catalysis is expanding for a variety of important applications. For instance, Nguyen and co-workers discussed the use of high-performing heterogeneous catalysts for the enhancement of the efficiency of Li–S batteries.^14^ Maneechakr et al., instead, reported the use of new sulfonic-magnetic activated carbon (S-MAC) catalysts to efficiently prepare 5-ethoxymethylfurfural (5-EMF), which is considered a highly sustainable biofuel, from the ethanolysis of biomass-based raw sugar.^15^

The catalytic performance is often enhanced by incorporating active sites within porous materials. This approach forces the reactants to stay in a highly restricted environment, such as the surface of a pore, thus maximizing their contact and, typically, accelerating the conversion rates of the products.^16,17^ Further improvements can be achieved when the active site is an integral part of the material (i.e., when it is chemically bound) as this significantly enhances catalyst reusability and mitigates the risk of leaching. An increasing number of examples of such materials are emerging, particularly for environmental applications.^18^ A particularly promising area of interest is the conversion of CO_2_ (often trapped to prevent its release into the environment) into added-value compounds.^19,20^ In a recent paper, Ji, Zhao, and Liu reviewed the progress of porous polymers to transform CO_2_ into fuels and chemicals,^21^ whereas Banerjee and his group reported the use of porous nanostructures for the efficient photocatalytic reduction of CO_2_ to CO in water.^22^

In the context of porous materials, polymers of intrinsic microporosity (PIMs) are arising as important platforms for important applications such as gas adsorption and separation,^23,24^ water purification,^25,26^ and desalination.^27^ PIMs owe their porosity to the rigid and contorted geometry of their monomers and the subsequent inefficient packing of their chains in the solid state, which produces pores of nanodimension.^28^ A few years ago a new type of polymerization based on the formation of Tröger’s base core (TB-PIMs) was introduced, which combines the typical high porosity of PIMs (i.e., the high BET surface area) with the presence of two basic nitrogen atoms per repeat unit.^29,30^ Despite the great potential for different applications, apart from selected examples, PIMs have not been much employed in catalysis.^31,32^ One of the factors that hampered their use in this field is attributed to the narrow size of their pores, which prevents the use of large substrates. In a recent study, our research team successfully addressed this issue by designing and synthesizing a series of TB-PIMs that can undergo swelling in the presence of a solvent, thereby expanding their pore sizes and facilitating the accommodation of larger reagents within their cavities. This alteration in morphology not only improved catalytic conversions but also broadened the range of compatible reagents, rendering these catalysts more versatile for general applications.^33^ Moreover, these materials exhibited exceptional stability and facile recyclability, which are largely attributed to the integration of the TB core within the polymeric backbone. These attributes are of paramount importance for the development of a good heterogeneous catalyst.^18,34^

In this study, we undertook the challenging, yet innovative task of designing monomers with geometries similar to those previously reported,^33^ to guarantee a swellable environment that facilitates the reactions and increases the conversions, but with a critical advancement: increasing the number of nitrogen atoms per repeat unit. This strategic modification proved to be crucial to enhance the catalytic performance of the materials by fine-tuning their basicity and nucleophilicity. The catalytic reaction of choice was the Knoevenagel condensation that, apart from being an important tool that helps produce value-added compounds,^35^ is perfectly suitable for the scope of this work as it is one of the most common protocols to test base catalysts and is highly comparable with our previous studies and from the literature.^33^ To demonstrate the increased basicity of the reported polymers, we extended the original Knoevenagel reaction, which typically uses benzaldehyde and malononitrile as the acidic proton source, to include other methylene species with lower proton acidity.

To further assess the improvements from a theoretical point of view, we applied quantum mechanical calculations to investigate how additional nitrogen atoms influence the basicity and enhanced performance of the reported polymers. In addition, a kinetic study was conducted to gain a deeper understanding of the substrate interactions with catalytic sites, providing valuable insights into the reaction mechanism.

## 2 Materials and Methods

Commercially available reagents and gases were used without further purification. All reactions using air/moisture-sensitive reagents were performed in oven-dried or flame-dried apparatus under a nitrogen atmosphere. TLC analysis refers to analytical thin-layer chromatography using aluminum-backed plates coated with Merck Kieselgel 60 GF254. Product spots were viewed either by the quenching of UV fluorescence or by staining with a solution of cerium sulfate in aqueous H_2_SO_4_. Melting points were recorded using a Cole-Parmer Stuart Digital Melting Point Apparatus and are uncorrected. Low-temperature N_2_ (77 K) and ambient CO_2_ (273 and 298 K) adsorption/desorption measurements of PIM powders were made using an Anton Paar Nova 600 BET surface area analyzer. Samples were degassed for 800 min at 80 °C under high vacuum prior to analysis. The data were analyzed with the software provided with the instrument. NLDFT analyses were performed to calculate the pore size distribution and volume, considering a carbon equilibrium transition kernel at 273 K based on a slit-pore model; the kernel is based on a common, one-center, Lennard-Jones model. TGAs were performed using the device PerkinElmer STA 6000 at a heating rate of 10 °C/min from 30 to 1000 °C. ^1^H NMR spectra were recorded in the solvent stated using an AVANCE Bruker DPX 500 (500 MHz) instrument, with ^13^C NMR spectra recorded at 125 MHz. Solid-state ^13^C NMR spectra were recorded using a Bruker AVANCE III spectrometer equipped with a wide-bore 9.4 T magnet (Larmor frequencies of 100.9 MHz for ^13^C). Samples were packed into standard zirconia rotors with a 4 mm outer diameter and rotated at a magic angle spinning (MAS) rate of 12.5 kHz. Spectra were recorded with cross-polarization (CP) from ^1^H using a contact pulse (ramped for ^1^H) of 1.5 ms. High-power (ν1 ≈ 100 kHz) TPPM-15 decoupling of ^1^H was applied during acquisition to improve resolution. Signal averaging was carried out for 6144 transients with a recycle interval of 2 s. Analysis of the polymer samples was carried out with a Phenom Pro X desktop SEM instrument (Phenom-World, Eindhoven, NL), equipped with a backscattering detector. The images of the cast powder morphology were acquired with an accelerating voltage of 10 kV.

All the details of each experiment, the synthesis of precursors, monomers, and polymers, and different figures, tables, and characterization can be found in the Supporting Information.

## 3 Results and Discussion

### 3.1 Synthesis of Monomers

**Figure 1:**
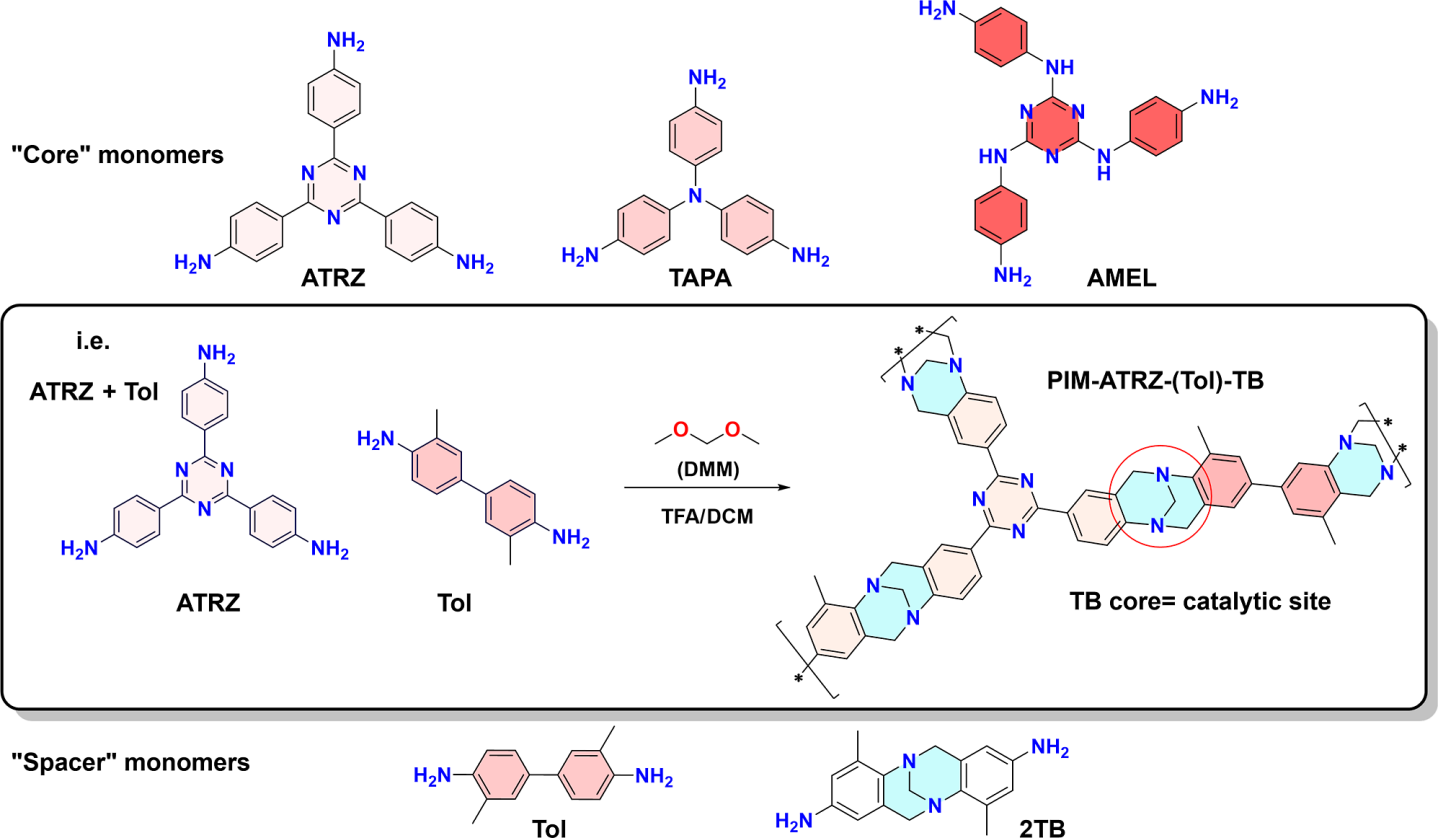
Synthesis of different polymers and copolymers (TFA = trifluoroacetic acid; DCM = dichloromethane; DMM = dimethoxymethane).

In our prior research on TB-PIMs for catalysis, we explored how the polymer chain swelling accelerates the catalytic reaction by making the active site more readily accessible. This effect is amplified when we introduce a solvent that further improves the swelling ability. In this work, we aim to show how the increase of the polymer’s basicity affects the catalytic properties of the materials while maintaining a similar level of swellability. To this end, we designed a series of polymers where a certain degree of free rotation in the backbone is allowed as we proved it to be crucial to improve the swellability, simultaneously adding groups that enhance the basicity/nucleophilicity of the catalytic sites.^33^ The latter is accomplished by introducing extra nitrogen atoms into the polymer’s framework, which has the potential to influence catalytic performance in two ways: (1) by adding extra basic groups, the TB core should become more nucleophilic; (2) by increasing the number of nitrogen atoms per repeating unit, more catalytic sites are available during the reaction. The second factor is the most prominent one as we anticipated that the electron density of the bridged TB nitrogen (so, its nucleophilicity) is difficult to be strongly influenced by functional groups that are relatively far from it. From this perspective, the most straightforward element that could affect the basicity of the polymer and, consequently, the catalytic conversions is the simple increase in the number of basic sites per repeating unit. The cores selected for our study are shown in Figure 1, and the overall synthesis is in Scheme S1.

We decided to start with a monomer that simply contains additional nitrogen, such as the triazine core of **ATRZ**. Despite its known electron-withdrawing nature, we anticipated that this heteroaromatic compound would enhance the attraction of polar reagents and stabilize the catalysis intermediates, along with promoting the proximity of the reactants to the active sites (TB).^36^ The second choice fell on the triaminophenylamine (**TAPA**), which features an extra tertiary amine per repeat unit that is expected to increase the overall basicity.^37,38^ The final core monomer we chose was triaminophenyltriazine (**AMEL**). Similar to ATRZ, it features the mildly electron-withdrawing triazine unit but, in this case, it is also connected to three nucleophilic secondary amines, which are supposed to introduce extra catalytic sites. Both triazine-based cores were already used to catalyze Knoevenagel-type reactions, although strongly activated by microwave irradiation at a temperature of around 80 °C.^39,40^ To further adjust the swellability and tune the overall nucleophilicity, we also opted to copolymerize these monomers with two “*spacers*”, namely, the commercial tolidine and synthetic Tröger’s base dianiline (**Tol** and **2TB** in Figure 1). The addition of the former is not expected to significantly alter the polymer’s basicity but, acting as a “*spacer*”, it should enhance the polymer’s swellability in the presence of a solvent, as demonstrated in our earlier work.^33^ The latter introduces an additional TB core that not only creates extra space between polymer chains but also effectively doubles the number of catalytic sites per repeat unit.

### 3.2 Synthesis and Characterization of Polymers

**Figure 2:**
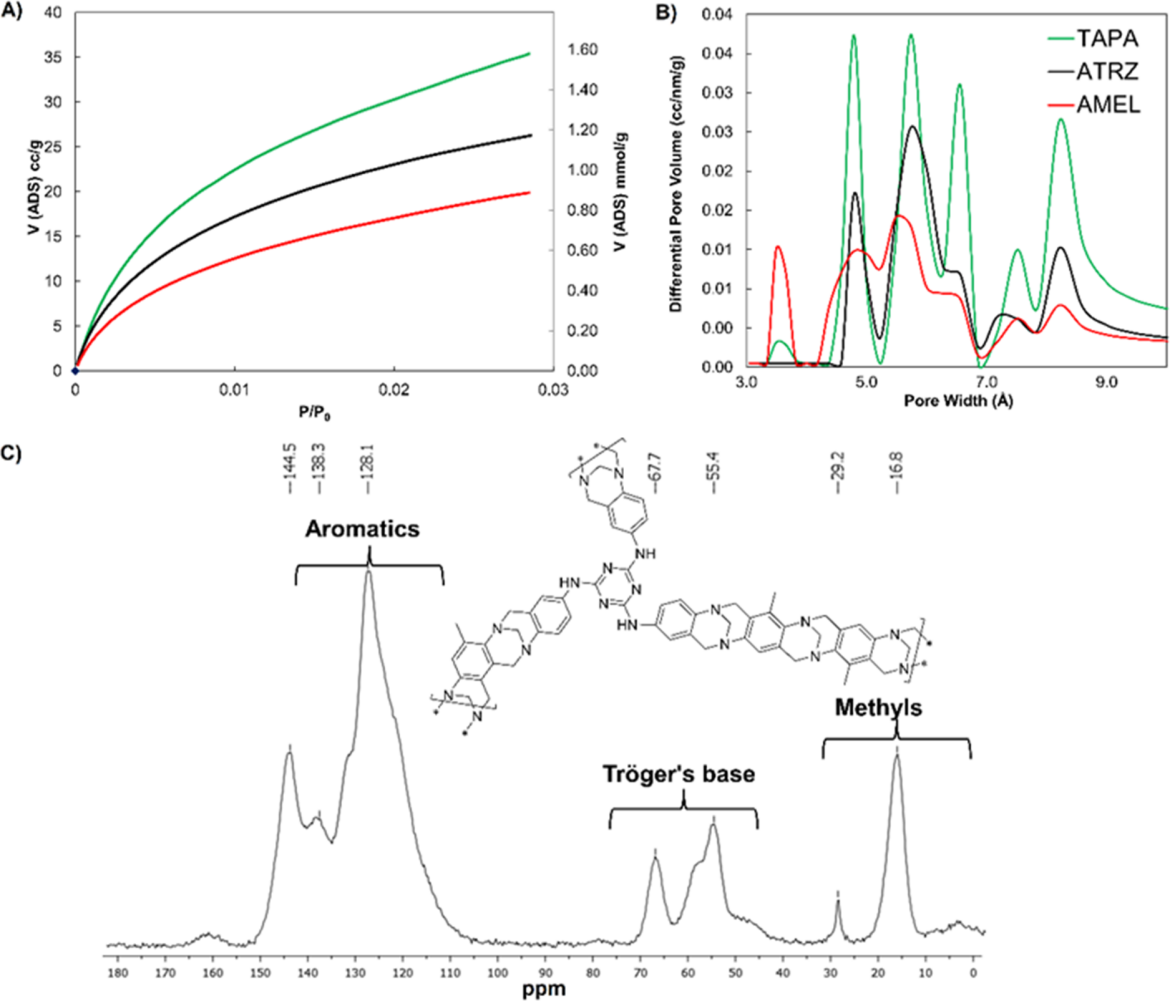
(A) CO_2_ adsorption isotherm at 273 K of selected PIMs; (B) Pore size distribution of PIMs; (C) ^13^C solid-state NMR of PIM-AMEL-(2TB).

The various core monomers were synthesized using established procedures (Scheme S1). ATRZ was produced through the cyclotrimerization of 4-aminobenzonitrile in the presence of triflic acid, a well-known method in triazine synthesis.^41^ Triaminophenylaminotriazine (AMEL) was prepared via the nucleophilic aromatic substitution of p-nitro aniline on cyanuric chloride, which is another typical method used to synthesize substituted triazines under mild conditions.^42,43^**TAPA** was simply obtained by reducing its correspondent nitro-version by reacting it with metallic tin in acidic media.^44^ Finally, the **2TB** “*spacer*” was produced from the reaction of 2-methyl-4-nitroaniline in trifluoroacetic acid (TFA), to create a TB monomer, which was followed by the reduction of its nitro groups to obtain the correspondent TB dianiline.^45^ Each polymerization was carried out under the same conditions previously developed by our research group.^29,33^ A representative example is given in Figure 1. Due to the hyperbranched nature of the polymers and their complete insolubility in common solvents, the confirmation of their molecular structures using solution-based techniques proved to be impossible, so successful polymerization was assessed by FT-IR and solid state ^13^C NMR (SSNMR) spectroscopy, as shown in Figure 2C for **PIM-AMEL-2TB** as a representative example (single spectra can be found in Figures S8–S15 for **NMR** and Figures S19–S21 for **FT-IR** spectroscopy). Especially, the latter feature shows that Tröger’s base core was effectively synthesized for all polymers as signals between 50 and 75 ppm are a typical signature of the methylene peaks of the TB core. Full details about each synthesis and characterization are given in the Supporting Information. The textural characterization of the materials showed that both BET surface area (SA_BET_) calculations and pore size distributions are similar for all synthesized compounds (Table 1 and Figures 2 and S1–S3). Since the slow kinetic of N_2_ at 77 K adsorption does not allow for a reliable measurement, the SA_BET_ was calculated from the isotherms of CO_2_ adsorption at 273 K and showed that the order of porosity was **PIM-TAPA-TB** > **PIM-ATRZ-TB** > **PIM-AMEL-TB**. These values cannot be deemed very accurate because of the known limitation of the BET method (especially when using CO_2_ as a probe gas as it is not the gas typically used in the BET model).^46,47^

**Table 1:** BET Surface Areas of PIM-TB Polymers

entry	polymer	monomer	co-monomer	SA_BET_^a^ (m^2^ g^–1^)	micropore volume^b^ (cm^3^ g^–1^)	CO_2_ uptake (mmol g^–1^)	decomposition temperature (°C)
1	PIM-ATRZ-TB	ATRZ		312	0.153	1.17	445
2	PIM-TAPA-TB	TAPA		380	0.159	1.58	440
3	PIM-AMEL-TB	AMEL		220	0.074	0.89	435
4	PIM-ATRZ-(Tol)-TB	ATRZ	Tol	220	0.099	1.10	440
5	PIM-ATRZ-(2TB)	ATRZ	TB	250	0.107	1.17	460
6	PIM-TAPA-(Tol)-TB	TAPA	Tol	260	0.135	1.48	420
7	PIM-TAPA-(2TB)	TAPA	2TB	270	0.109	1.28	440
8	PIM-AMEL-(2TB)	AMEL	2TB	285	0.125	1.40	440

a Calculated from CO_2_ (273 K).

b Narrow micropore volume calculated from the Dubinin–Radushkevich equation, calculated from CO_2_ adsorption at 273 K.

It seems that the more rigid structure of TAPA provides the highest porosity of the set, while the two triazine-containing networked PIMs show a reduced surface area. The polymer containing AMEL has the lowest porosity of the entire series, which is not surprising considering the free rotation site around the extra secondary amine, allowing it to pack more efficiently in the solid state, thus reducing the free volume. The results also correlate with the micropore volume obtained via the Dubinin–Radushkevich equation, calculated from CO_2_ adsorption at 273 K. We observed that copolymers with an additional TB core introduce an extra “arm” to the material. Consequently, the BET surface area of a homopolymer (e.g., TAPA-TB) is slightly higher than its counterpart with the extra arm (2TB). In the case of PIM-AMEL-(2TB), which is slightly more porous than the corresponding homopolymer, we note that the monomer itself has a free rotation site that typically reduces the porosity by allowing more efficient packing. However, in this case, in contrast with the other copolymers, the addition of the extra TB core may make it slightly more rigid, producing a slight increase of its porosity.

Considering that the structures of these polymers are very similar, and also that we used the same method developed in our previous work, we can safely assume the accuracy of the trend of porosity shown in Table 1. The moderate porosity and the broad pore size distribution (PSD) suggest high swellability in the presence of a solvent, a factor that significantly enhanced catalytic performance in our previous study.^33^ The CO_2_ adsorption isotherms (273 K, Figure 2A) confirm that the more porous polymers adsorb slightly higher amounts of this probe gas, which is expected. This method was preferred over the adsorption of N_2_ at 77 K (normally used to calculate BET surface areas) as the latter produced uneven isotherms that hint at a very slow adsorption kinetic. The fact that the polymers display porosity when using CO_2_ as a probe gas suggests that the pores “*open up*” with the increase of the temperature, and this is due to an enhanced motion of the polymeric chains that confirms the good swellability (see the Supporting Information for more details).

The analysis of the PSD (Figures 2B and S3) shows very similar pore sizes for all of the obtained polymers. Since in our previous work we demonstrated that there is not a strict correlation between surface area and catalytic performance, this was not cause for concern, although we also proved that a certain degree of porosity has an influence on the conversions.^33^

**Figure 3:**
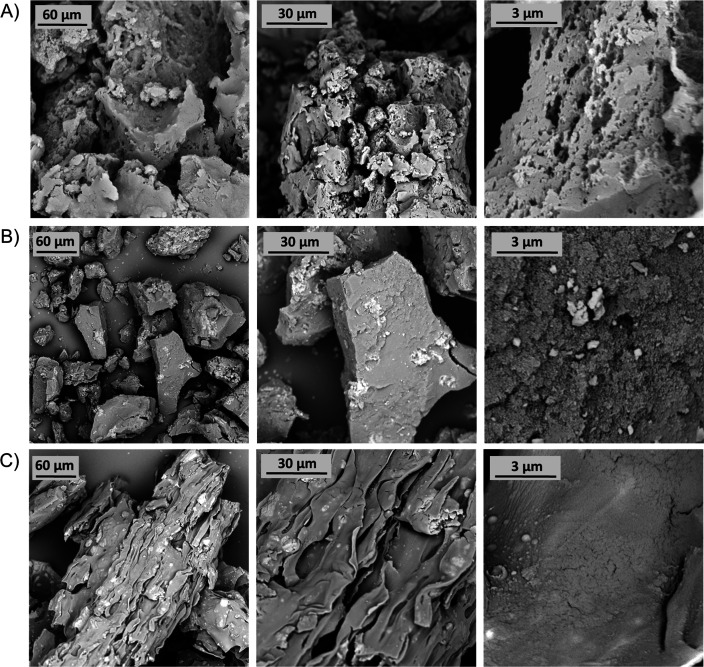
SEM images of (A) PIM-TAPA-TB; (B) PIM-AMEL-TB; (C) PIM-AMEL-(2TB).

Considering that we intentionally engineered these materials to exhibit uniform swellability levels, to be sure that the comparison of catalytic performance was minimally influenced by this factor, the remarkable similarity in both SA_BET_ and PSD can be regarded as beneficial for the study. Thermal studies of the three main polymers (namely, ATRZ, AMEL, and TAPA) were conducted by thermal gravimetric analysis (TGA) and are reported in Figure S18, showing that all polymers are very stable with an order **PIM-ATRZ-TB** > **PIM-TAPA-TB** > **PIM-AMEL-TB** with decomposition temperatures between 435 and 445 °C. To assess the morphology of the polymers and copolymers, we acquired SEM images (Figure 3).

The qualitative evaluation of the micrographs indicates that the surface of **PIM-TAPA-TB** is slightly rougher compared to that of **PIM-AMEL-TB**, which appears smoother. This observation aligns with the BET surface area measurements (Table 1) as the former exhibits higher porosity and is expected to be less swellable, whereas the latter, being less porous due to the extra “*free rotation*” sites given by the AMEL core, results in a denser and smoother surface. Additionally, to check for differences between polymers and copolymers, we examined the morphology of PIM-AMEL-(2TB), which proved to be slightly smoother than the corresponding homopolymer. This supports the hypothesis that the addition of extra TB “arms” further enhances the swellability. These observations are particularly evident at higher magnifications, such as in the images at 3 μm in Figure 3.

### 3.3 Catalytic Properties of Polymers

#### 3.3.1 Knoevenagel Condensation of Benzaldehydes and Malononitrile

To compare the current results with our previous work, the same kind of Knoevenagel condensation was employed to assess all of our polymers. The investigation started with the solvent-free reaction of benzaldehyde and malononitrile in a 3:1 stoichiometric ratio. As possible to assess from Table 2, the current set of polymers did not exhibit superior performance under these conditions. In fact, the best performing material proved to be “old” TAPB-PIM, which achieved approximately 95% conversion within one hour.^33^ This could be due to the slightly lower surface areas of these PIMs in comparison to their predecessors. Indeed, despite the presence of additional basic sites, the reduced surface area likely played a significant role in the performance. This is in line with our observation that more flexible polymers experience reduced catalytic turnovers in the absence of a “swelling” solvent. This phenomenon can be ascribed to the contraction of the polymer chains, which effectively restricts access to some “buried” catalytic sites, thus forcing the catalysis to happen primarily on the exposed surface of the material. The addition of an extra TB core in **PIM-TAPA-(2TB)** slightly improved the performance, most likely due to the addition of an extra basic site per repeat unit that brings the conversion almost on par with our previously published best polymers (entries 11 and 12 in Table 2). We also report turnover numbers (TON) and turnover frequencies (TOF) for all catalyzed reactions.

**Table 2:**
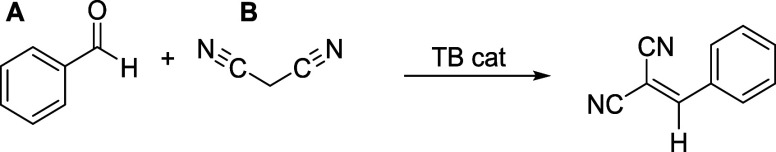
Conversions of Knoevenagel Reaction—3:1 Benzaldehyde/Malononitrile in Solvent-Free Conditions^a^

entry	catalyst	conversion (%) at varying times (mins)					at maximum conversion		at 20 min	
		20	40	60	80	120				
	polymers						TON^b^ (mol mol^–1^)	TOF^c^ (h^–1^)	TON^b^ (mol mol^–1^)	TOF^c^ (h^–1^)
1	PIM-ATRZ-TB	51	66	76	85	93	62	31	34	103
2	PIM-TAPA-TB	44	70	83	86	90	60	30	29	89
3	PIM-AMEL-TB	58	72	84	90	98	**65**	**33**	**39**	**117**
4	PIM-TAT-TB^32^	45	63	74	80	93	62	31	30	91
5	PIM-TAPB-TB^33^	64	85	94	97		**64**	**48**	**43**	**130**
	copolymers									
6	PIM-ATRZ(Tol)-TB	22	56	72	82	89	45	23	11	33
7	PIM-ATRZ-(2TB)	77	88	92	93	96	32	16	26	77
8	PIM-TAPA(Tol)-TB	63	84	92	96	100	50	35	32	95
9	PIM-TAPA-(2TB)	69	92	97	99		33	20	23	70
10	PIM-AMEL-(2TB)	22	38	49	60	77	26	13	7	22
11	PIM-TAPB + A1-TB^33^	86	100				50	76	43	130
12	PIM-TAPBext + A1-TB^33^	95	100				50	76	48	144
13	no catalyst				30					
14	TB homogeneous	25	35	54	65	85	85	43	25	76

a Experimental conditions: 3 mmol of benzaldehyde, 1 mmol of malononitrile, 1 mol % TB-catalyst.

b Turnover number after 20 min and at maximum conversion, calculated from no. of moles of malononitrile consumed versus no. of mole equivalents of TB catalyst.

c Turnover frequency calculated from turnover number per hour (or mol nitrile per mol catalyst per hour, mol mol^–1^ h^–1^).

Table 2 shows the values at maximum conversions and after 20 min, which allows a full comparison with the best-performing polymers that achieved complete conversion within just 20 min. From these results, we found that under solvent-free conditions and with a 3:1 benzaldehyde/malononitrile ratio, **PIM-AMEL-TB** is the best performing polymer of the new series. It achieved a TON of 65 at maximum conversion and 33 after 20 min. These values are extremely close to those of our previously reported materials.^33^

#### 3.3.2 Knoevenagel Reaction in Ethanol

**Figure 4:**
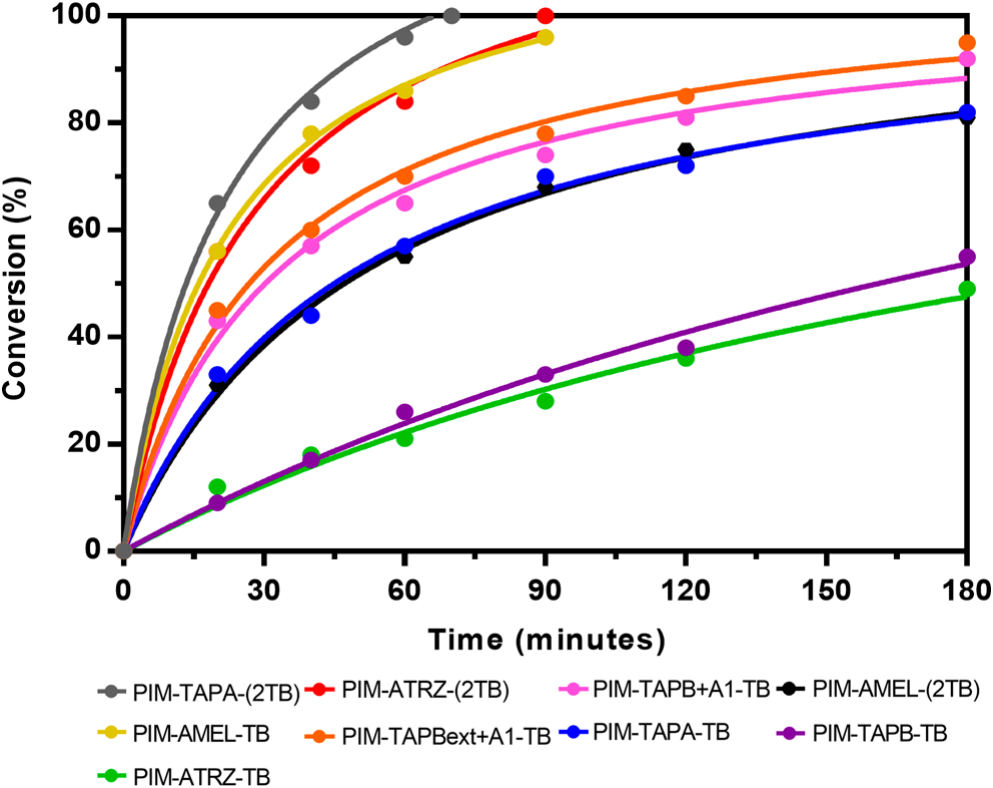
Conversions in the Knoevenagel reaction—1:1 4-*tert*-butyl-benzaldehyde: malononitrile in ethanol (fitted with Prism as hyperbola or sigmoidal 4PL both least-squares, 99%).

As expected, a substantial enhancement in performance was observed for all polymers when the reaction was conducted in the presence of ethanol as a “swelling solvent” and employing a 1:1 ratio of malononitrile/benzaldehyde, as shown in Table 3. A number of polymers within this series yielded results on par with our top-performing PIMs,^32,33^ with the most effective materials achieving complete conversion within 20 min. **PIM-AMEL-TB** displayed the highest TON and TOF (67 and 202). This marks the best result not only among the current series but also compared to our previously reported work, further confirming the improvements achieved with this study.^33^ Given the already outstanding performance exhibited in our prior research, it could be postulated that the enhanced properties are due to the contribution of both the swelling effect and the increased number of basic sites. However, with the simple combination of the small benzaldehyde and malononitrile, there is no room for significant improvement. Subsequently, the same polymers were employed as catalysts for the reaction between the bulkier 4-*tert*-butyl-benzaldehyde and malononitrile, in a 1:1 ratio and with ethanol as the swelling solvent. Remarkably, several TB-PIMs achieved conversions exceeding 95% within 90 min or less (**PIM-TAPA-(2TB)** in 60 min and **PIM-AMEL-TB** in 90 min), which is approximately one-third to half of the time it took the best performing polymers of our previous work PIM-TAPB + A1-TB and PIM-TAPBext + A1-TB, in similar conditions (Figure 4 and Table S1).

**Table 3:**
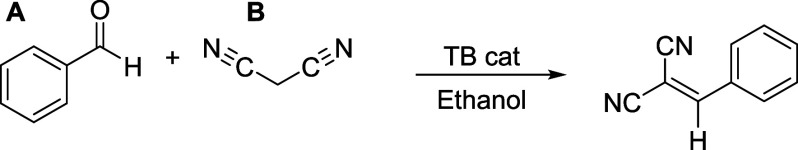
Conversions of the Knoevenagel Reaction—1:1 Benzaldehyde: Malononitrile in Ethanol at 25 °C^a^

entry	catalyst	conversion (%) at varying times (mins)					at maximum conversion		at 20 min	
		10	20	30	40	60	TON^b^ (mol mol^–1^)	TOF^c^ (h^–1^)	TON^b^ (mol mol^–1^)	TOF^c^ (h^–1^)
1	PIM-ATRZ-TB	48	65	80	100		67	101	43	131
2	PIM-TAPA-TB	46	66	74	100		67	101	44	133
3	PIM-AMEL-TB	71	100				**67**	**202**	**67**	**202**
4	PIM-ATRZ(Tol)-TB	17	33	44	61	73	37	37	17	50
5	PIM-ATRZ-(2TB)	80	100				33	100	33	100
6	PIM-TAPA(Tol)-TB	7	15	23	31	41	21	21	7.5	23
7	PIM-TAPA-(2TB)	70	92	100			33	67	31	93
8	PIM-AMEL-(2TB)	75	94	100			33	67	31	95
10	PIM-TAPB-TB	64	95	98	100		67	101	63	192
11	PIM-TAPB + A1-TB^33^	64	87	95	100		50	75	44	132
12	PIM-TAPBext + A1-TB^33^	88	100				50	151	50	151

a Experimental conditions: 1 mmol of benzaldehyde, 1 mmol of malononitrile, 1 mol % TB-catalyst.

b Turnover number after 20 min and at maximum conversion, calculated from no. of moles of malononitrile consumed versus no. of mole equivalents of TB catalyst.

c Turnover frequency calculated from turnover number per hour (or mol nitrile per mol catalyst per hour, mol mol^–1^ h^–1^).

It is worth noting that, with *t*Bu-benzaldehyde, **PIM-TAPA-(2TB)** and **PIM-AMEL-TB** emerged as the top-performing PIMs in *solvent-free* conditions, yet they achieved only 32% and 28% conversion, respectively (see Figure S4). This further confirms the substantial improvement in the catalytic activity when a solvent is introduced into the system. Given that the BET calculations and pore size distribution data (Table 1 and Figure 2) suggest that these polymers exhibit a very similar degree of flexibility between each other and compared to the best polymers of our previous work (plausibly attributed to an increased level of free rotation around specific chemical bonds), the superior performance of the reported materials must be ascribed to the increased number of catalytic sites.

Undoubtedly, this enhancement is a direct result of the enhanced polymer design reported in this study. Moreover, it is crucial to highlight that **PIM-AMEL-TB** stands out as the most effective polymer that does not bear an additional TB, rising as the best performing core of the set. Recyclability tests were conducted with **PIM-AMEL-TB** using benzaldehyde and malononitrile 1:1 in ethanol, as a typical run. In total, six cycles were run with no significant loss of catalytic activity. More details are in Figure S17. After recycling the catalysts, we tested their stability in both aqueous NaOH and 6N H_2_SO_4_ at temperatures up to 60 °C. We found that the polymers remained very stable under these conditions and maintained the same activity. In the typical Knoevenagel reaction mechanism, where malononitrile and benzaldehyde are involved, it is the malononitrile that interacts with the base catalyst, generating a carbanion intermediate after the abstraction of one of its α-protons. This subsequently attacks the carbonyl group of the aldehyde to form the mixture of carbinol first and dehydrated species later.^48,49^ Up to now, our investigations have primarily focused on varying the aldehyde species to assess the accessibility of the catalytic sites, which is correlated with polymer porosity and swellability; therefore, we also decided to change the methylene species.

#### 3.3.3 Changing the α-Proton-Containing Methylene Species

In the case of these nitrogen-rich polymers, it is also essential to assess the influence that their enhanced basicity has on the reagents directly affected by the catalysts (i.e., the methylene species). We examined this aspect by replacing malononitrile with two similar cyanoacetates and a cyanoacetamide, which allowed us to gain deeper insights into the catalyst’s relative reactivity. The first attempt was conducted with methyl cyanoacetate (monomer B, Table 4), inspired by its use in previous works.^50,51^ To fine-tune the reaction conditions for this new reactant, we employed **PIM-TAPA-TB**, which showed midrange performance among the catalysts tested in our prior experiments. In a standard reaction, benzaldehyde and methyl cyanoacetate were mixed in a 3:1 ratio under solvent-free conditions, replicating the initial conditions we always used for our Knoevenagel tests. The mixture was stirred at room temperature with 1 mol % of the **PIM-TAPA-TB** catalyst. The first effort only achieved a 7% conversion after 6 h. The reaction was then repeated using a 1:1 ratio of methyl cyanoacetate to benzaldehyde, this time also adding ethanol as the typical swelling solvent. Between 3 and 6 h at room temperature, we obtained only 30% conversion. Consequently, understanding that more time was needed compared to the reaction with malononitrile, the reaction was allowed to stir overnight, which resulted in a conversion rate of 72%. It became evident that the conditions that had previously proven effective for malononitrile were inadequate for obtaining efficient yields with the cyanoacetate core. To improve the performance, we raised the temperature up to 50 °C employing both 1:1 and 3:1 molar ratios of benzaldehyde and obtaining the best results when an excess of benzaldehyde was used.

**Table 4:**
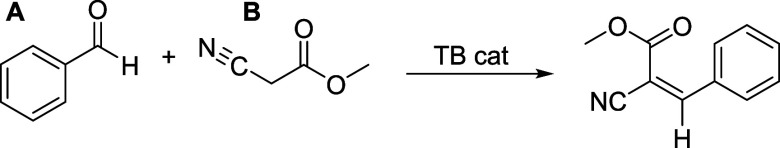
Optimized Conditions for the Reaction between Benzaldehyde (A) and Methyl Cyanoacetate (B) Using 1 mol % of PIM-TAPA-TB as a Catalyst^a^

entry	molar ratio of reagents (A/B)	solvent	temperature (°C)	conversion (%)		at maximum conversion	
				6 h	18 h	TON^b^ (mol mol^–1^)	TOF^c^ (h^–1^)
1	3:1		25	7			
2	1:1	EtOH (2 mL)	25	30	72	48	2.7
3	1:1	EtOH (2 mL)	50	45	80	53	2.9
4	1:1	water 2 mL)	50	43	84	56	3.1
5	1:1	EtOH (4 mL)	**50**	**62**	**84**	**56**	**3.1**
6	3:1	EtOH (2 mL)	**50**	**83**	**100**	**67**	**3.7**
7	3:1	EtOH (2 mL)	25	52	86	57	3.2

a Experimental conditions: 1 mmol of benzaldehyde, 1 mmol of methyl cyanoacetate, 1 mol % TB-catalyst.

b Turnover number at maximum conversion, calculated from no. of moles of methyl cyanoacetate consumed versus no. of mole equivalents of TB catalyst.

c Turnover frequency calculated from turnover number per hour.

**Figure 5:**
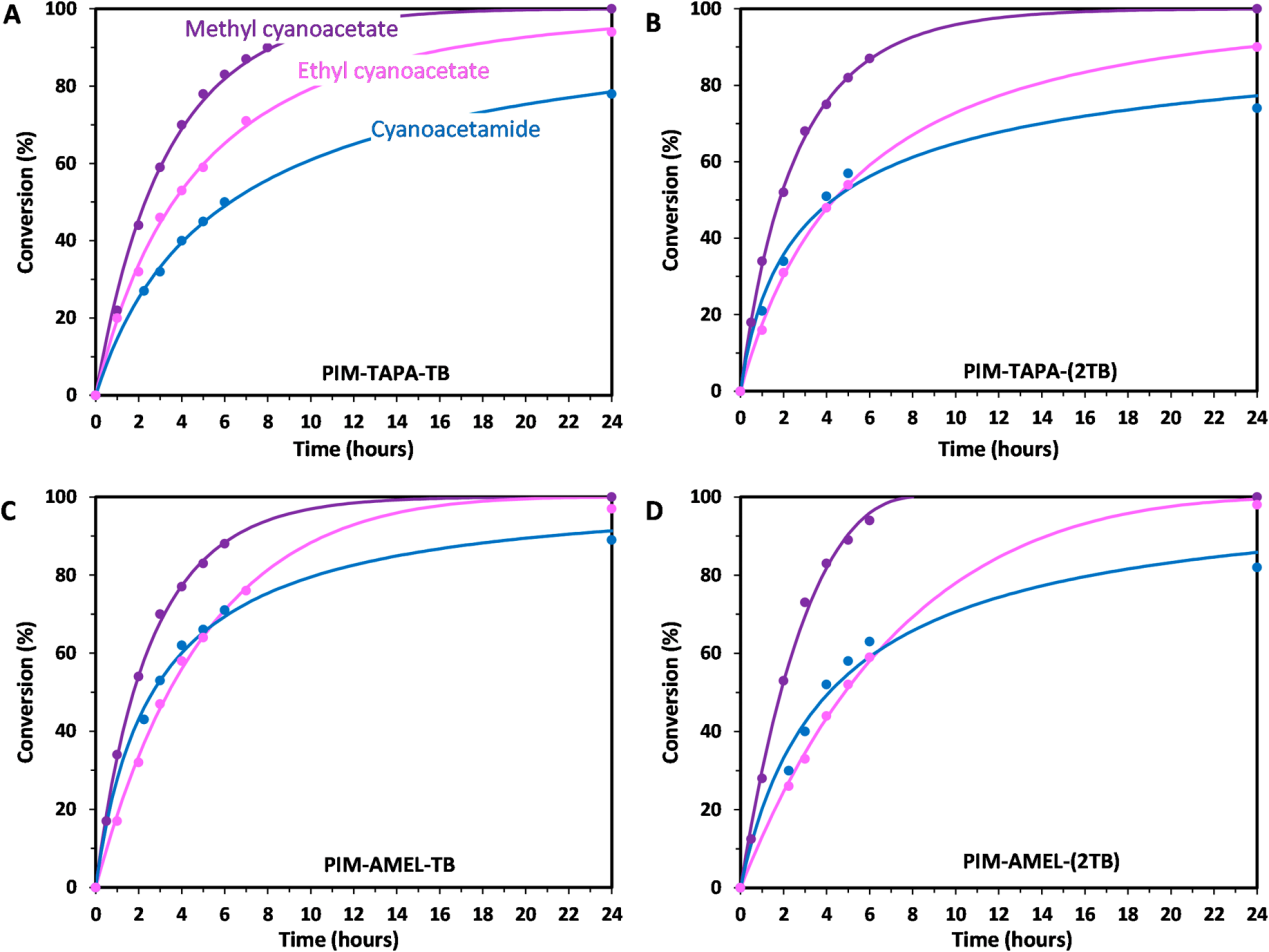
Performance comparisons with varying methylene species. (A) PIM-TAPA-TB; (B) PIM-TAPA-(2TB); (C) PIM-AMEL-TB; (D) PIM-AMEL-(2TB). Lines represent a fit of the data with a kinetic model (see Section 3.5).

Under these conditions, the highest conversion achieved full completion in 18 h (Table 4, entry 6), but, upon closer examination and hourly sampling, it was revealed that the reaction had reached completion in just 10 h, yielding a remarkable 83% conversion after only 6 h, as shown in Table S2 and Figure 5. The reaction conducted with a 3:1 ratio of benzaldehyde but at room temperature (25 °C), outperformed the 1:1 attempt, yet it only achieved 86% conversion after 18 h (with 52% conversion during the initial 6 h, Figure S6). Based on these results, we established that the optimum conditions for using cyanoacetate corresponded to **entry 6** in Table 4, which are 50 °C, 3:1 stoichiometry benzaldehyde/malononitrile, and ethanol (although also the 1:1 ratio performed reasonably well). We then ran a systematic analysis of the other polymers and copolymers in these conditions (Figure S5), where it became apparent that both **AMEL-TB**-containing polymers stood out as the top-performing materials, as also confirmed by its TON and TOF, achieving a remarkable 93% conversion within 6 h in the case of **PIM-AMEL-(2TB)**.

This outcome is not unexpected as this polymer possesses the greatest number of basic sites per repeating unit. It is worth highlighting that despite the fact that all of the studied polymers share very similar features, we anticipated that the inclusion of triazine (ATRZ) would not significantly enhance the catalytic performance. Indeed, all three polymers featuring the simple **ATRZ** were found to be less active than the others. This could be attributed to the weakly basic nature of its core, which leads to just partial stabilization of the intermediates with consequent loss of activity.^52^

### 3.4 Computational Studies

**Figure 6:**
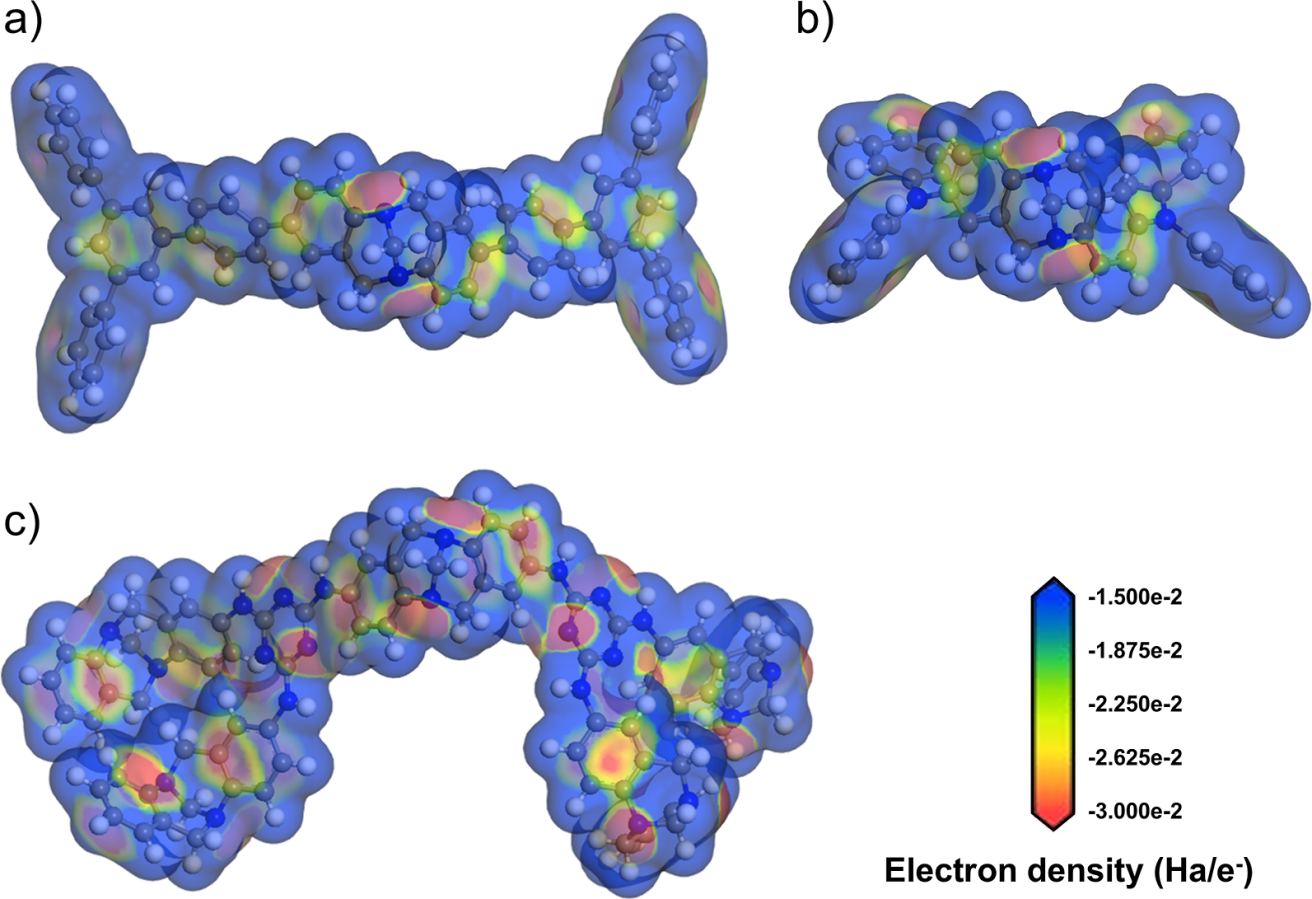
Electrostatic potential maps landscapes of (a) TAPBext-PIM; (b) PIM-TAPA; and (c) PIM-AMEL-TB. The quantification of electron density is expressed in Hartrees per electron (Ha/e^−^).

**Figure 7:**
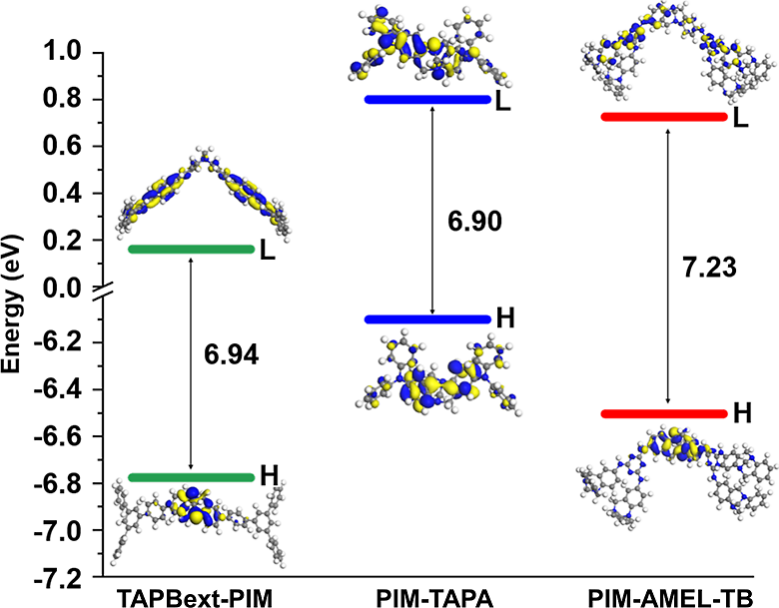
Molecular orbital diagrams and calculated HOMO–LUMO energy levels for TAPBext-PIM, PIM-TAPA, and PIM-AMEL-TB. (Legend: L is LUMO (lowest unoccupied molecular orbital) and H is HOMO (highest occupied molecular orbital). Density functional theory using NWChem 7.0.2 software.^53^ The geometries were optimized, allowing the relaxation of the entire structures, using the CAM-B3LYP functional.^54^

The trend in the change of reactivity of these polymers was confirmed by quantum mechanical studies, which show that the basicity of the repeat unit of **PIM-AMEL**, **PIM-TAPA**, and **TAPBext-PIM**(33) depends mainly on the nitrogen of the TB-cores and those of the amines that can freely share their lone pair, rather than the ones involved in the aromatic ring of the triazine (Figures 6a–c and S16). The analysis of the molecular orbitals of TAPBext-PIM (Figure 7 and Table S3) suggests that the highest occupied molecular orbital (HOMO) is predominantly situated over the TB core, whereas the lowest unoccupied molecular orbital (LUMO) spans the benzene rings as well as the TB unit.

For **PIM-TAPA**, the distribution of both HOMO and LUMO is quite uniform, extending across the entire molecule and incorporating both the TB and TAPA units. In the case of **PIM-AMEL-TB**, the HOMO is primarily localized on the TB, and the LUMO is confined to the molecule’s triazine units. The energy gap between the HOMO and LUMO indicates the chemical reactivity and polarizability of these molecules. Among them, **PIM-TAPA** exhibits the smallest energy gap (Δ*E*_HOMO–LUMO_ = 6.90 eV), and **PIM-AMEL-TB** exhibits the highest (Δ*E*_HOMO–LUMO_ = 7.23 eV).

**PIM-TAPA-TB** and **PIM-TAPA-(2TB)** also showed good performance, achieving conversions exceeding 80% within the same 6 h time frame. This reactivity is primarily attributed to the TAPA units, which allow the lone pairs of nitrogen atoms to participate in electronic delocalization, enhancing the molecule’s polarizability. The highest HOMO value observed in **PIM-TAPA** (−6.10 eV) highlights the molecule’s electron-donating capability. Conversely, the lowest LUMO value found in **TAPBext-PIM** (0.16 eV) highlights its electron-accepting capacity. The variation in energy gaps underscores the unique electronic properties of these molecules, with the TAPA units significantly influencing the molecular reactivity through electronic delocalization, while the aromatic triazine components contribute to the stability of the **PIM-AMEL-TB** molecule. Although these values can be considered very similar, the general interpretation is that **PIM-TAPA** shows higher catalytic activity because of the more reactive TB-core, whereas **PIM-AMEL-TB** shows improvements because of the increased number of N-sites.

The electrostatic potential landscapes mapped on the van der Waals surfaces of **PIM-AMEL-TB**, **PIM-TAPA**, and **TAPBext-PIM** (Figure 6a,c) show distinct zones of negative and positive potentials. The positive potential regions, marked by a blue hue, are predominantly associated with the outer hydrogens of aromatic rings, suggesting areas of lesser electron density. The red zones denote negative potentials, where the highest electron concentration is located. As expected, they are primarily localized around the electron-rich nitrogen of Tröger’s base, and the areas influenced by its strong electron-donating properties. For **TAPBext-PIM**, the presence of a singular Tröger’s base unit is mirrored in the red zones, marking areas of significant electron concentrations (Figure 6a). In the case of **PIM-TAPA**, the negative potential zones are proximal to one TB unit and two TAPA units, as visualized in Figure 6b. The electron density distribution within **PIM-AMEL-TB** is attributed to the incorporation of TB units, the other secondary amines that also contribute to the catalysis, and the triazine units, as evidenced in Figure 6c.

From the modeling studies, we can conclude that the electronic configuration of the TAPA core enhances the nucleophilicity (and so the catalytic activity) of the repeat unit due to the electron donating effect that the tertiary amine has on the TB core, whereas the improved activity of **PIM-AMEL-TB** is simply due to the higher number of basic sites, compared to **TAPBext-PIM**. The optimized geometries for the polymers and the surface electrostatic potential (ESP) results are shown in Tables S4–S6. To conclude our study, the four most promising polymers (**AMEL** and **TAPA**) were assessed for the condensation of benzaldehyde with methyl cyanoacetate under *room temperature* conditions over the same time span of the higher temperature tests (Figure S6). All of them exhibited very similar performance, each achieving approximately 50% conversion within the initial 6 h period, which can be deemed as a very good result considering the known lower reactivity of methyl cyanoacetate compared to malononitrile, which often requires activation such as microwave irradiation and ionic liquids.^55−57^ Finally, to assess the effectiveness of our optimized method, we employed AMEL and TAPA polymers as catalysts, utilizing other methylene sources, namely, ethyl cyanoacetate and cyanoacetamide.^58,59^ Each polymer showed only slightly different conversions with these new reagents over a 6 h time period, but over 24 h, the ethyl version of cyanoacetate proved to be a better reagent than the corresponding acetamide. This outcome is in line with expectations, given the reduced acidity of acetamide’s α-protons: the general reactivity of these methylene sources follows the trend malononitrile (p*K*_a_ ∼ 11) > cyanoacetate (p*K*_a_ ∼ 13) > cyanoacetamide (p*K*_a_ ∼ 17).^60^ The trend can be explained by the mesomeric effect of the amide that reduces the acidity of the neighboring α-protons, making the species less nucleophilic and thus less prone to attack the carbonyl of the aldehyde. However, even the poorly reactive acetamide showed nearly equivalent performance compared to the esters, possibly helped by its smaller size, which offsets the lower acidity. Consistently throughout our catalytic tests, methyl cyanoacetate outperformed its ethyl counterpart, achieving full conversion in 24 h and attaining 50–60% within the initial 6 h. The best outcomes with cyanoacetamide were achieved with the two **PIM-AMEL-TB** polymers, which showed 82–89% conversions in 24 h, with 50–65% conversion within the first 6 h (as seen in Figure 5 and detailed in Table S2).

### 3.5 Comparison with Other Studies

Our study demonstrated comparable, and often superior, performance to similar polymers reported in the literature. For instance, Luan et al. reported conversions between 87 and 99% within 2–5 h using benzaldehyde/malononitrile (1/1.5) and amino-modified MOFs as catalysts, but they had to use toluene as the reaction solvent.^61^ Zarei and co-workers developed a triazine-urea porous polymer (so, very similar to AMEL), achieving 95% conversions in 30–60 min with various benzaldehydes, but requiring temperatures up to 100 °C.^62^ Liu et al. designed a pillar[5]ene-Tröger’s base material to catalyze Knoevenagel condensations and for the cycloaddition of CO_2_ to epoxides to form cyclic carbonates, attaining 87% conversions using ethanol as a solvent over 4 h at 80 °C.^63^ Machado and co-workers achieved 96% conversions in the Henry reaction using a triazene-linked porous organic polymer (also very relevant for this work) with nitro-benzaldehyde and nitromethane at 60 °C in 6 h.^64^ Hou et al. prepared polymers with triphenyl benzene moieties, reaching 98% in 3 h using benzaldehyde/malononitrile at room temperature in methanol as the solvent.^65^ Lastly, Rodríguez-Gonzalez et al. developed Tröger’s base polymers that achieved 98% conversion for the condensation of benzaldehyde and malononitrile with 10% molar catalysts in 24 h.^66^

### 3.6 Reaction Kinetics

**Figure 8:**
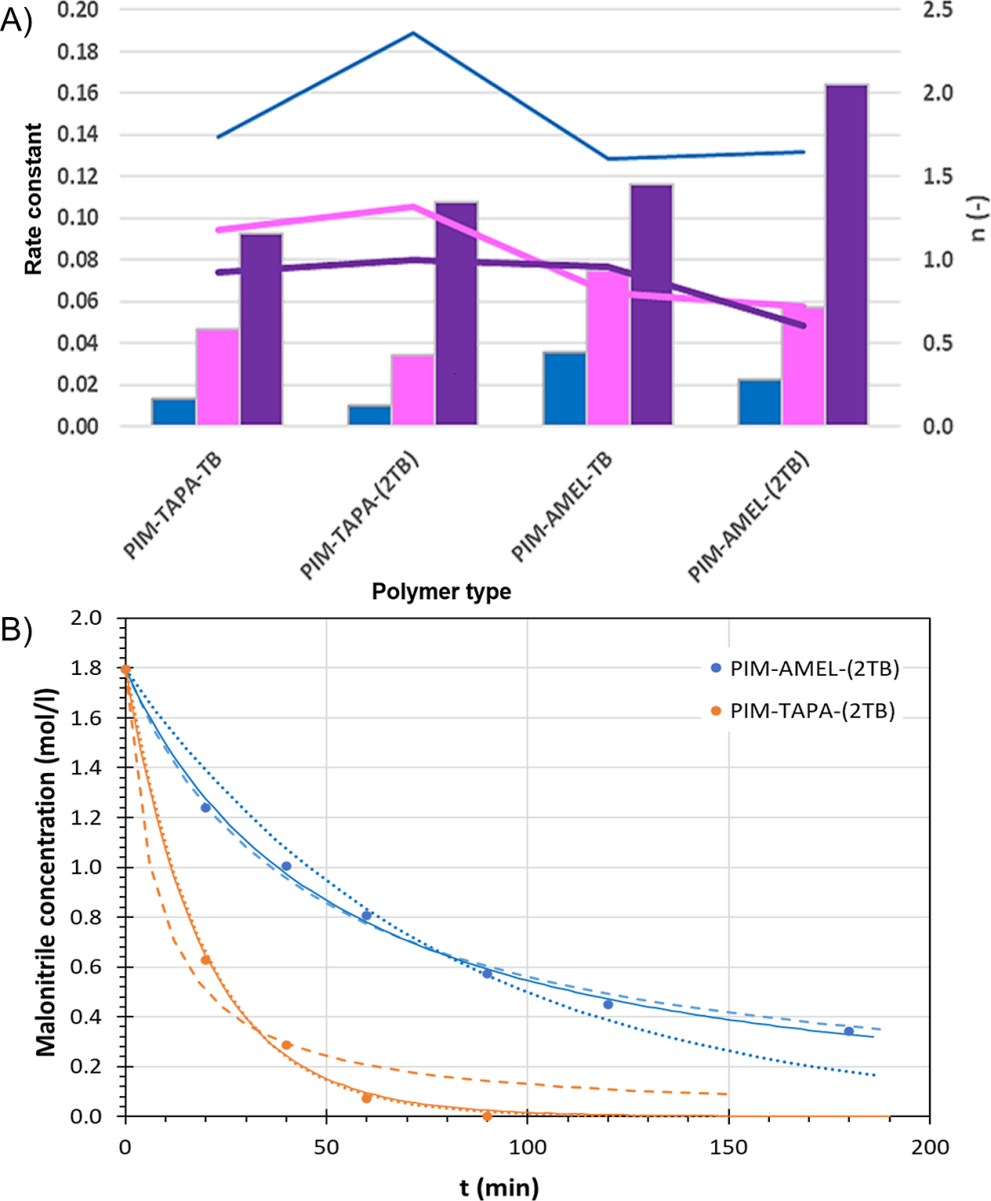
(A) Fitting parameters of the data in Figure 5 with the model  with the assumption *n* = *m*. Bars indicate the rate constant at the left axis and lines indicate the reaction order at the right axis (blue = cyanoacetamide; pink = ethyl cyanoacetate; purple = methyl cyanoacetate). (B) Fit of the malononitrile concentration in the reaction with 4-*tert*-butyl benzaldehyde with a first-order reaction (SEq. 6, dotted line ······) or second-order reaction in malononitrile (SEq. 9, dashed line - - - - -), and with *n*th order reaction in both malononitrile and 4-*tert*-butyl benzaldehyde (SEq. 1 or SEq. 13, thin continuous line—) using PIM-AMEL-(2TB) and PIM-TAPA-(2TB).

Under normal circumstances, if there are no rate-limiting intermediate steps or other phenomena, like diffusion limitations of the reagents or products to or from the catalytic site, and if both reactants are involved in the rate-limiting step, then the reaction is expected to be first order in the nitrile and the aldehyde^67^
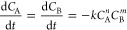
1where *C*_A_ and *C*_B_ are the concentrations of the nitrile and the aldehyde, *k* is the reaction rate constant, and *n* and *m* are both equal to 1. In more complex situations,^68^ the rate-determining step may involve only one of the two reactants (*n* = 0 or *m* = 0), or there may be other phenomena related, for instance, to diffusion limitations or to very high substrate affinity, resulting in a different reaction order, with *n*, *m* < 1 if the reactant concentration at the catalytically active site is lower, or *n*, *m* > 1 if it is higher than that in the bulk. Herein we fitted the results of Figure 5 with eq 1 to perform a preliminary study of the reaction kinetics for a better understanding of the trends. Its integral cannot be solved analytically to give a simple equation for the concentration profile versus time, but the profile can be obtained by numerical integration and a least-squares estimation of the parameters *k*, *n,* and *m* (see Supporting Information Section S5 for further explanation). In our example, both methyl cyanoacetate and ethyl cyanoacetate fit very well with this model, and the reaction order is close to 1 (Figure 8A), as predicted for the first-order reaction in both reagents. The substantially higher rate constants for **PIM-AMEL-(2TB)** and to a lesser extent in **PIM-AMEL-TB** seem to be associated with a somewhat lower reaction order, which might suggest diffusion limitations of the reactants to the catalytically active site. Instead, the reaction of cyanoacetamide seems slower and fits well only for **PIM-TAPA-TB**, but with the three other catalysts, it shows faster conversions in the first 5 h (Figure S7) and then rapidly slows down to reach the lowest rate of all after 24 h. This trend cannot be described by any of the common reaction kinetics models (Supporting Information, Section S5), which suggests some kind of activity decline of the catalyst or simply that, after a few hours, the formation of more solid product blocks the pores slightly inhibiting the further conversion of the remaining reactants. Given that even the kinetics of homogeneous catalysis in the Knoevenagel reaction are nontrivial,^67,69^ the need to conduct a more comprehensive investigation to enhance our comprehension of its behavior under heterogeneous conditions seems imperative, especially with highly porous materials where a better assessment of the diffusion factors could be crucial. Detailed studies with different ratios of the reagents are needed for a complete understanding of this phenomenon but are outside the scope of the present work.

The catalyst has an even stronger impact on the reaction kinetics than the reagents, not only in terms of reactivity but also in terms of reaction mechanism. Figure 8B shows the concentration of malononitrile as a function of time during the reaction in ethanol with an equimolar amount of 4-*tert*-butyl benzaldehyde, using **PIM-AMEL-(2TB)** and **PIM-TAPA-(2TB)** as the catalysts. The reaction catalyzed by **PIM-AMEL-(2TB)** can be described very well by a second-order decay of the malononitrile concentration. This is in practice equivalent to a first-order reaction in both the nitrile and the aldehyde since both have the same concentration and the reaction is stoichiometric. Indeed, the best fit with eq 1 yields *n* = *m* = 0.88, close enough to 1 to be within the experimental error. Interestingly, despite its slightly lower BET surface area (270 m^2^ g^–1^ for **PIM-TAPA-(2TB)** versus 285 m^2^ g^–1^ for **PIM-AMEL-(2TB**)), (Table 1), the reaction with **PIM-TAPA-(2TB)** is not only much faster but also follows first-order kinetics in malononitrile, indicating that the rate-limiting step most likely involves only one of the two reagents. With equimolar amounts of the nitrile and the aldehyde, this experiment cannot distinguish which of the two reagents is involved in the rate-limiting step, but it shows the power of the kinetics experiments to provide fundamental information on the reaction. A more detailed analysis of different reagent ratios could identify the precise role of each substrate and will be the subject of future work.

## 4 Conclusions

In this work, we have demonstrated the ease of functionalization of PIMs for heterogeneous catalysis, especially focusing on how these modifications affect the reactivity of the acidic species involved in the catalytic process. The design of these materials allowed us to add additional catalytic sites, leading to increased performance in the Knoevenagel reaction between malononitrile and substituted benzaldehydes. To prove the validity of our methods, the polymer catalysts were also tested with other acidic methylene-containing species, and we found that the polymers with the AMEL cores performed best. This was attributed to the presence of more basic nitrogen, especially considering the presence of secondary amine sites that may improve the overall activity. TAPA-cored polymers also performed very well, also because of the presence of an extra tertiary amine in the center of the core, while ATRZ polymers underperformed in comparison to their other counterparts. Despite our attempts, it proved impossible to assess any differences in the p*K*_a_ of each polymer, so we based our considerations merely on the increased number of basic sites, which attack the methylene species more promptly and make them available for the nucleophilic attack on the benzaldehyde’s carbonyls. This is supported by the calculation of the electrostatic potential maps of representative polymers showing similar values for the TB cores but different basic sites in the repeat units. We can safely claim that the combination of enhanced basicity/higher number of nitrogen and the tuned swellability of these materials can significantly affect the reaction kinetics and effectively improve the catalytic properties of Tröger’s base PIMs. Kinetic studies finally provide useful insight into the possible cause of changes in the reaction rate and may thus help in the design of more effective systems.

## Supporting Information Available

Synthesis and characterization of monomers and polymers; adsorption studies; tables; various figures; and solid-state ^13^C NMRs (PDF)
