# Supplementary material for: Nitrogen Enriched Tröger’s Base Polymers of Intrinsic Microporosity for Heterogeneous Catalysis
Source: ACS Appl Polym Mater. 2024 Dec 18;7(1):220–33. doi: 10.1021/acsapm.4c02952 (PMC11730871; doi:10.1021/acsapm.4c02952)
Supplement: Supplementary file 1 — ap4c02952_si_001.pdf [file ap4c02952_si_001.pdf]

Supporting Information

Nitrogen Enriched Tröger's Base Polymers of Intrinsic Microporosity (TB-PIMs) for Heterogeneous Catalysis.

Natasha Hawkins,[a] Ariana R. Antonangelo,[a] Mitchell Wood,[a] Elena Tocci,[b] Johannes Carolus Jansen,[b] Alessio Fuoco,[b] Carmen Rizzuto,[b] Mariagiulia Longo,[b] C. Grazia Bezzu[a] and Mariolino Carta[a]*

[a] Department of Chemistry, Faculty of Science and Engineering, Swansea University, Grove Building, Singleton Park, Swansea, SA2 8PP, UK.

*Email: mariolino.carta@swansea.ac.uk

[b] Institute on Membrane Technology, National Research Council of Italy (CNR-ITM), via P. Bucci 17/C, Rende (CS), 87036, Italy

General methods and equipment....S2
Experimental section....S3
1. Synthesis of monomers....S3
2. Synthesis of polymers....S6
3. Gas adsorption analysis....S11
4. General catalysis test:....S12
5. Reaction kinetics....S15
6. ^{13}C Solid State NMR....S18
7. Computational Studies....S22
8. Recyclability test....S32
9. TGA curves....S33
10. FT-IR....S34
References....S35

General methods and equipment

Commercially available reagents and gases were used without further purification. All reactions using air/moisture sensitive reagents were performed in oven-dried or flame-dried apparatus, under a nitrogen atmosphere. TLC analysis refers to analytical thin layer chromatography, using aluminium-backed plates coated with Merck Kieselgel 60 GF254. Product spots were viewed either by the quenching of UV fluorescence, or by staining with a solution of Cerium Sulfate in aqueous H_2SO_4. Melting points were recorded using a Cole-Parmer Stuart™ Digital Melting Point Apparatus and are uncorrected. Low-temperature N_2 (77 K) and CO_2 (273 K and 298 K) adsorption/desorption measurements of PIM powders were made using an Anton Paar Nova600. Samples were degassed for 800 min at 80 °C under high vacuum prior to analysis. The data were analysed with the software provided with the instrument. NLDFT analyses were performed to calculate the pore size distribution and volume, considering a carbon equilibrium transition kernel at 273 K based on a slit-pore model; the kernel is based on a common, one centre, Lennard-Jones model. TGAs were performed using the device PerkinElmer STA 6000 at a heating rate of 10 °C/min from 30 to 1000 °C. ^{1}H NMR spectra were recorded in the solvent stated using an Avance Bruker DPX 500 (500 MHz) instruments, with ^{13}C NMR spectra recorded at 125 MHz. Solid-state ^{13}C NMR spectra were recorded using a Bruker Avance III spectrometer equipped with a wide-bore 9.4 T magnet (Larmor frequencies of 100.9 MHz for ^{13}C). Samples were packed into standard zirconia rotors with 4 mm outer diameter and rotated at a magic angle spinning (MAS) rate of 12.5 kHz. Spectra were recorded with cross polarisation (CP) from 1H using a contact pulse (ramped for 1H) of 1.5 ms. High-power ($\nu_1 \approx$ 100 kHz) TPPM-15 decoupling of 1H was applied during acquisition to improve resolution. Signal averaging was carried out for 6144 transients with a recycle interval of 2 s. Chemical shifts are reported in ppm relative to $(CH_3)_4Si$ (TMS) using the CH_3 signal of L-alanine (δ = 20.5 ppm) as a secondary solid reference.

Experimental section

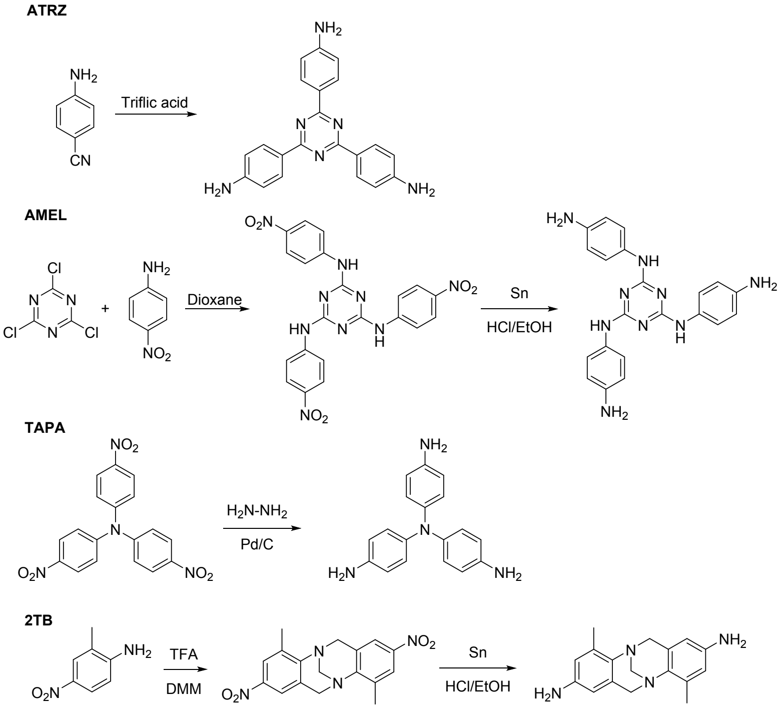

Scheme S1. Synthesis of the various precursors and monomers.

1. Synthesis of monomers

Triaminophenyltriazine (ATRZ)[1]

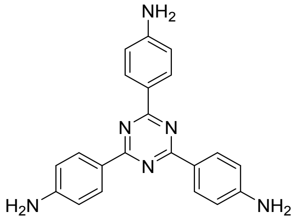

4-aminobenzonitrile (3.00 g, 25.4 mmol) was stirred in trifluoromethanesulfonic acid (7.76 mL, 85.7 mmol) under a nitrogen atmosphere for 24 hours at room temperature. The reaction was poured into water (50 mL) and neutralised with NaOH. The crude product was filtered and washed with portions of water. The product was then dried to give a bright yellow solid. (2.58 g, 86 %). MP: >300 °C; IR: ν_{max} (cm^{-1}) 3461, 3378, 3320, 3210, 3030, 1633, 1605, 1576, 1492, 1430, 1364, 1294, 1178, 1148, 955, 851, 810, 642, 589, 512; ^{1}H NMR: (500 MHz; DMSO-d_6) δ 8.34 (d, 6H, ArH), 6.68 (d, 6H, ArH), 5.9 (s, 6H, NH); ^{13}C NMR: (125 MHz; DMSO-d_6) δ 167.6, 153.5, 130.6, 122.3, 113.8; MS: calculated $C_{21}H_{18}N_6$ 356.17 found 354.9 [M^+].

Trinitrophenylaminotriazine (TNMEL)[2]

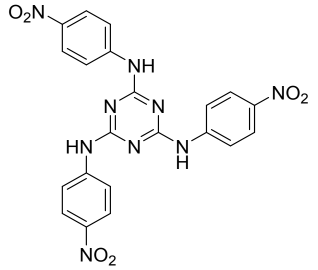

Cyanuric chloride (2.00 g, 10.8 mmol) was stirred to 1,4-dioxane (120 mL), followed by *p*-nitroaniline (7.47 g, 54.2 mmol) and K_2CO_3 (9.00 g, 65.1 mmol). The mixture was then refluxed at 100 °C for 24 hours. The crude product was filtered, washing with portions of cold water and methanol. The product was dried under vacuum to produce a pale-yellow solid. (4.28 g, 81%). MP: >300 °C; IR: ν_{max} (cm^{-1}) 3334, 3085, 1624, 1589, 1518, 1481, 1420, 1295, 1244, 1186, 1108, 1010, 846, 796, 749, 686, 493, 456; ^1H NMR: (500 MHz; DMSO-d_6) δ 10.25 (s, 3H, NH), 8.20 (d, 6H, ArH), 8.07 (d, 6H, ArH) ppm; ^{13}C NMR: (125 MHz; DMSO-d_6) δ 147, 125, 124, 120, 119; MS: calculated $C_{21}H_{15}N_9O_6$ 489.11 found 489.5 [M^+].

Triaminophenylaminotriazine (AMEL)[3, 4]

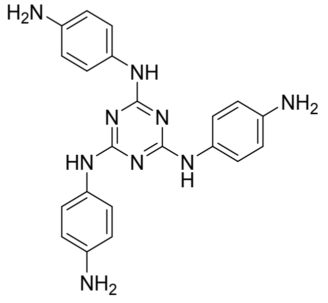

2,4,6-Tris(*p*-nitrophenylamino)-1,3,5-triazine (2.80 g, 5.72 mmol) was stirred in a mixture of ethanol (50 mL) and hydrochloric acid (50 mL). Tin (6.15 g) was added, and the mixture was refluxed at 80 °C for 48 hours. The resulting reaction mixture was filtered to remove the solvent, and the crude solid product was dissolved in hot water. The mixture was filtered, and the filtrate was basified using NaOH solution to a pH of 11. The solid product was collected washed with water and methanol, to give a pale-yellow powder. (1.05 g, 46%). MP: 285 °C; IR: ν_{max} (cm^{-1}) 3446, 3363, 2807, 2571, 2106, 1620, 1556, 1494, 1418, 1340, 1249, 1178, 1113, 1067, 1014, 886, 826, 748, 511; ^1H NMR: (500 MHz; DMSO-d_6) δ 8.48 (s, 3H, NH), 7.33 (s, 6H, ArH), 6.50 (s, 6H, ArH) 4.74 (s, 6H, NH), 3.73 (s, 6H) ppm; ^{13}C NMR: (125 MHz; $CDCl_3$) δ 162.0, 145.0, 137.0, 118.0, 114.0; MS: calculated $C_{21}H_{21}N_9$ 399.19 found 399.2 [M^+].

4,10-dimethyl-2,8-dinitro-6H,12H-5,11-methanodibenzo[b,f][1,5] diazocine (TB-NO_2)[5]

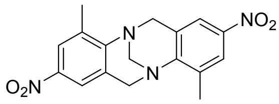

To a mixture of 2-methyl-4-nitroaniline (10.00 g, 65.8 mmol) TFA (80 mL) in an ice bath, paraformaldehyde (4.77 g, 160 mmol) was gradually added. The ice bath was removed, and the reaction was left to stir at room temperature for 14 days. The reaction mixture was poured into water and basified to pH 9 with NaOH. The crude product was collected by filtration and resuspended in hot acetone for 20 minutes. The solution was cooled, and then placed in the freezer at -20 °C for 16 hours. The solid product was collected and dried to give a yellow solid. (10.18 g, 91%). MP: >300 °C; IR: ν_{max} (cm^{-1}) 3393, 1677, 1586, 1508, 1436, 1329, 1290, 1206, 1140, 1098, 1067, 974, 950, 894, 794, 760, 746, 650, 522, 494; ^1H NMR: (500 MHz; DMSO-d_6) δ 7.97 (s, 2H,), 7.82 (s, 2H,), 4.68 (d, 2H,), 4.36 (s, 2H,), 4.33 (d, 2H,), 2.48 (s, 6H, CH_3) ppm; ^{13}C NMR: (125 MHz; DMSO-d_6) δ 153.1, 143.5, 135.3, 129.8, 124.0, 121.3, 67.6, 55.5, 17.1; MS: calculated $C_{17}H_{16}N_4O_4$ 340.12 found 339.9 [M^+].

4,10-dimethyl-6H,12H-5,11-methanodibenzo[b,f][1,5]diazocine-2,8-diamine (2TB)[5]

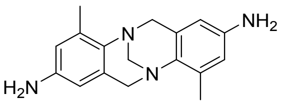

4,10-dimethyl-2,8-dinitro-6H,12H-5,11-methanodibenzo[b,f][1,5] diazocine (4.50 g, 13.2 mmol) was stirred in a mixture of ethanol (100 mL) and hydrochloric acid (100 mL). Tin (15.77 g) was added, and the reaction was refluxed at 80 °C for 24 hours. The reaction was filtered, and the filtrate was neutralised with NaOH. Then crude product was collected and washed in DCM. The product was then filtered and dried to give an off-white solid. (3.5 g, 94%). MP: >300 °C; IR: ν_{max} (cm^{-1}) 3316, 2951, 2335, 2098, 1914, 1613, 1478, 1323, 1214, 1012, 918, 846, 513; ^1H NMR: (500 MHz; $CDCl_3$) δ 6.82 (s, 2H, ArH), 6.53 (s, 2H, ArH), 5.33 (s, 2H, NCH_2), 4.28 (s, 4H, NCH_2), 3.45 (br s, 4H, NH), 2.36 (s, 6H, CH_3) ; ^{13}C: NMR (125 MHz; $CDCl_3$) δ 142.2, 138.6, 134.5, 129.9, 116.5, 110.7, 68.6, 55.9, 17.5; MS: calculated $C_{17}H_{20}N_4$ 280.17 found 280.8 [M^+].

Triaminophenylamine (TAPA)[6]

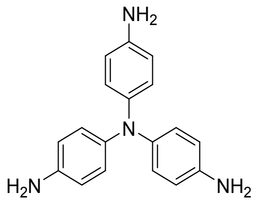

Tris(nitrophenyl)amine (3.04 g, 8.0 mmol) was stirred in a mixture of Pd/C (0.20 g) and ethanol (150 mL). Hydrazine monohydrate (16 mL, 323.9 mmol) was added dropwise over a 30-minute period. The reaction was then heated to 80 °C and refluxed overnight. The reaction was then hot filtered and washed with hot ethanol. The solvent was removed by rotary evaporation and then the crude product was recrystallised in ethanol. The mixture was placed in the freezer at -20 °C overnight, and then the product was collected and dried to give fine silver crystals. (1.68 g, 72%) MP: 240 °C; IR: ν_{max} (cm^{-1}); ^1H NMR: (500 MHz; DMSO-d_6) 6.6 (d, 6H, ArH), 6.46-6.43 (d, 6H, ArH), 4.70 (br s, 6H, NH); ^{13}C NMR: (125 MHz; DMSO-d_6) 143.5, 139.5, 124.6, 115.3 MS: calculated 290.15 $C_{18}H_{18}N_4$ found 291.1 [M^+].

2. Synthesis of polymers

General Procedure A

All polymers were prepared according to literature procedures of PIM synthesis,[7] with some modifications where necessary. A chosen tri-substituted monomer (1 molar equivalent) was reacted with dimethoxymethane (DMM) (7-8 equivalents) in DCM (approximately 6-12 mL), followed by dropwise addition of trifluoroacetic acid (TFA) (37 equivalents). The reaction was left to stir at room temperature until the mixture was viscous, where it was crashed out in a mixture of ammonia and ice and stirred overnight. The product was then filtered, washed with plenty of water, and refluxed in acetone, THF, DCM and methanol for 1h each, and then refluxed in methanol one further time overnight. The polymer was then dried in a vacuum oven at 85 °C for 24 hours.

General Procedure B

All polymers were prepared according to literature procedures of PIM synthesis with modifications.[7] Two chosen monomers (in varying molar ratios depending on number of polymerisation sites*) was reacted with dimethoxymethane (DMM) (7-8 equivalents) in DCM (approximately 6-12 mL), followed by dropwise addition of trifluoroacetic acid (TFA) (37 equivalents). The reaction was left to stir at room temperature until the mixture was viscous or jelly-like, typically 16 hours. The resulting reaction was ground down into fine parts, and then poured into a mixture of ammonia and ice and stirred overnight. The product was then filtered, washed with plenty of water, and refluxed in acetone, THF, DCM and methanol for 1h each, and then refluxed in methanol one further time overnight. The polymer was then dried in a vacuum oven at 85 °C for 24 hours.

* For a combination of two di-substituted monomers, or two tri-substituted monomers, they are combined in a 1:1 molar ratio. For a combination of one di- and one tri-substituted monomers, they are combined in a 3:2 molar ratio, to ensure complete polymerisation at all sites.

PIM- AMEL-TB

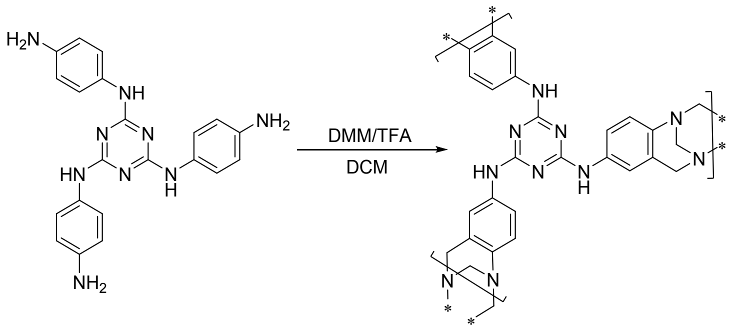

General procedure A was followed using tris(aminophenyl)aminotriazine (AMEL) (1.00 g, 2.50 mmol) and dimethoxymethane (0.6 mL, 17.6 mmol) were stirred in DCM (10 mL), followed by dropwise addition of TFA (7.08 mL, 92.6 mmol). The reaction was left to stir for 18 hours, to yield a brown solid. (1.13 g, 84% yield) BET: (CO_2, 273 K) = 220 m^2 g^{-1}; Total pore volume = 0.03 (at P/P_0 ~ 0.98); TGA: initial mass loss at 435 °C; FT-IR: ν max (cm^{-1}) 3370, 2105, 1567, 1481, 1403, 1225, 1198, 1061, 962, 922, 803, 512.

PIM-ATRZ-TB

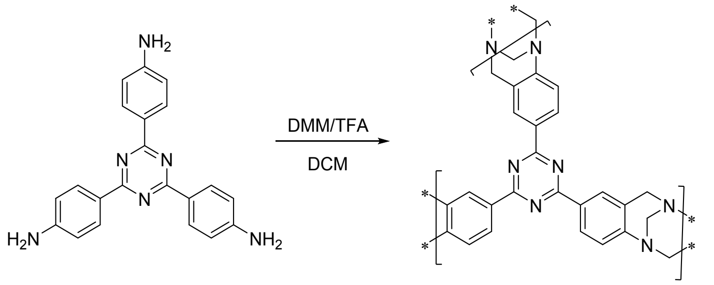

General procedure A was followed using tri(aminophenyl)triazine (ATRZ) (0.50 g, 1.41 mmol) and dimethoxymethane (0.4 mL, 11.3 mmol) were stirred in DCM (8 mL), followed by dropwise addition of TFA (4.9 mL, 64.1 mmol). The reaction was left to stir for 16 hours, to yield a yellow solid. (0.58 g, 86% yield) BET: (CO_2, 273 K) = 312 m^2 g^{-1}; Total pore volume = 0.05 (at P/P_0 ~ 0.98); TGA: initial mass loss at 445 °C; FT-IR: ν max (cm^{-1}) 3660, 2973, 2883, 1676, 1603, 1497, 1357, 1183, 944, 815, 578.

PIM-AMEL-(2TB)

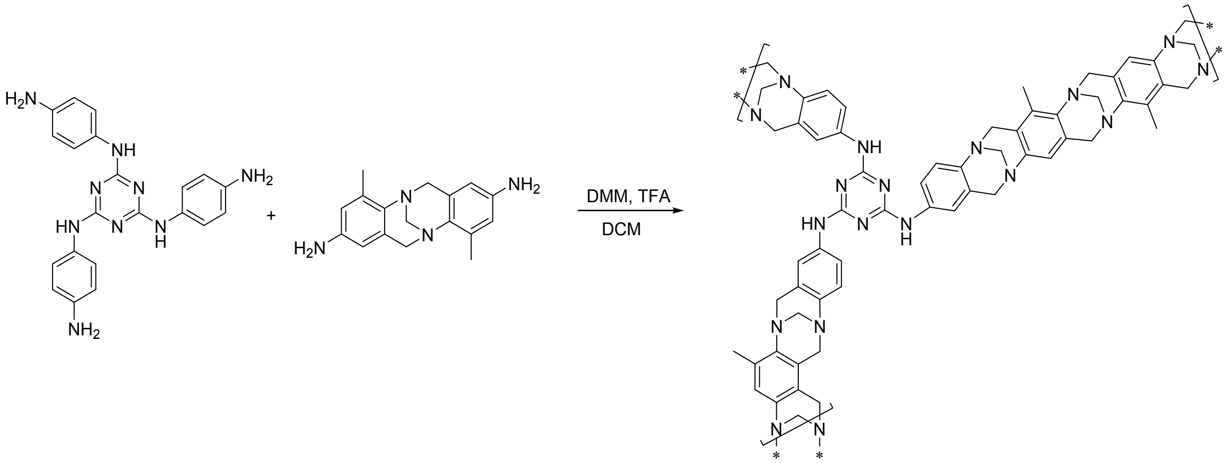

General procedure B was followed using tri(aminophenyl)aminotriazine (AMEL) (0.65 g, 1.63 mmol), 2TB (0.68 g, 2.44 mmol) and dimethoxymethane (1.01 mL, 11.41 mmol) were stirred in DCM (12 mL), followed by dropwise addition of TFA (5.0 mL, 62.8 mmol). The reaction was left to stir for 16 hours, to yield a pale-yellow solid. (1.12 g, 77% yield) BET: (CO_2, 273 K) = 285 m^2 g^{-1}; Total pore volume = 0.04 (at P/P_0 ~ 0.98); TGA: initial mass loss at 440 °C; FT-IR: ν max (cm^{-1}) 3663, 2922, 1981, 1708, 1610, 1486, 1265, 1207, 1065, 925, 829, 826, 720, 608, 529, 436.

PIM-ATRZ-(2TB)

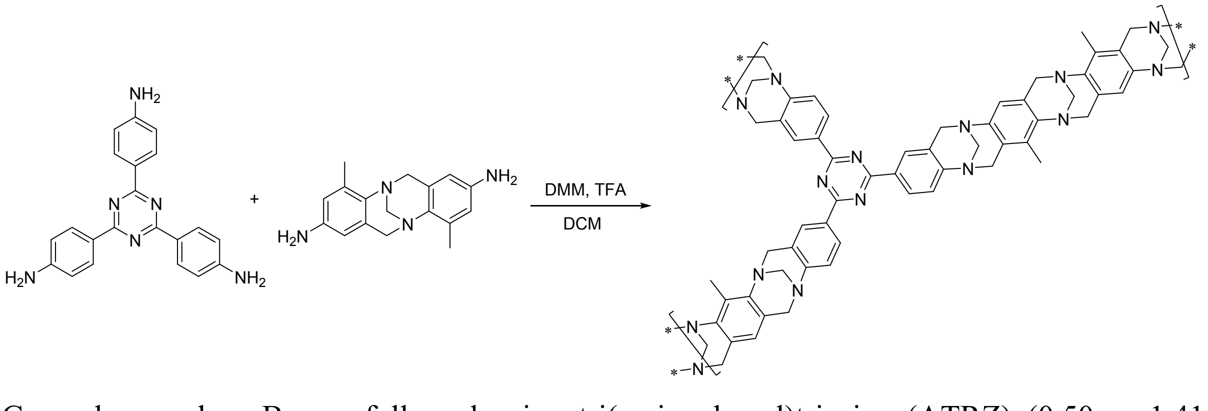

General procedure B was followed using tri(aminophenyl)triazine (ATRZ) (0.50 g, 1.41 mmol), 2TB (0.59 g, 2.12 mmol) and dimethoxymethane (0.9 mL, 10.2 mmol) were stirred in DCM (12 mL), followed by dropwise addition of TFA (4.0 mL, 52.3 mmol). The reaction was left to stir for 16 hours, to yield a pale-yellow solid. (0.95 g, 69% yield) BET: (CO_2, 273 K) = 250 m^2 g^{-1}; Total pore volume = 0.04 (at P/P_0 ~ 0.98); TGA: initial mass loss at 460 °C; FT-IR: ν max (cm^{-1}) 3399, 2900, 1672, 1605, 1502, 1416, 1358, 1285, 1181, 1144, 813, 534.

PIM-ATRZ-(Tol)-TB

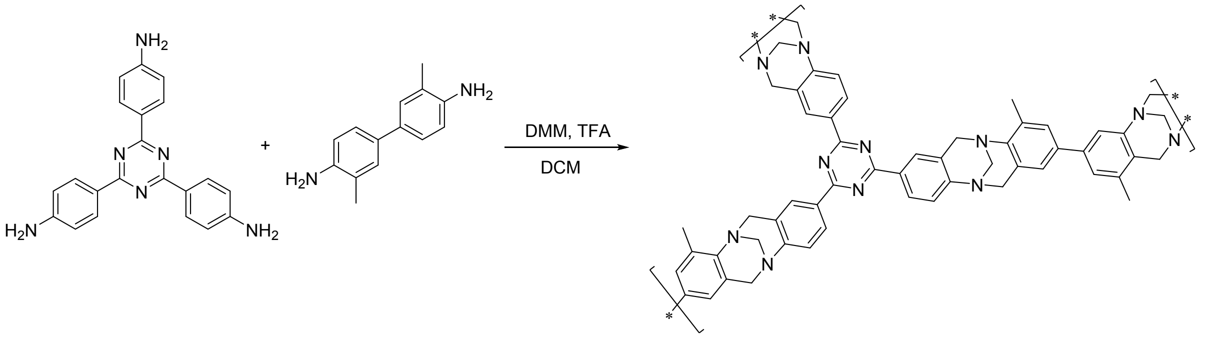

General procedure B was followed using tri(aminophenyl)triazine (ATRZ) (0.61 g, 1.72 mmol), tolidine (Tol) (0.55 g, 2.58 mmol) and dimethoxymethane (1.22 mL, 13.77 mmol) were stirred in DCM (10 mL), followed by dropwise addition of TFA (5.93 mL, 77.4 mmol). The reaction was left to stir for 16 hours, to yield a pale-yellow solid. (1.04 g, 71%) BET: (CO_2, 273 K) = 220 m^2 g^{-1}; Total pore volume = 0.03 (at P/P_0 ~ 0.98); TGA: initial mass loss at 440 °C; FT-IR: ν max (cm^{-1}) 2920, 2109, 2019, 1667, 1604, 1503, 1351, 1177, 810, 510, 416.

PIM-TAPA-TB

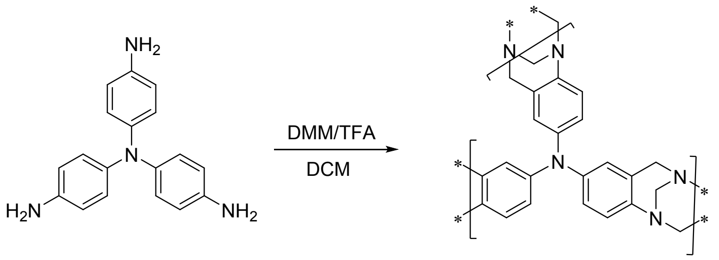

General procedure A was followed using extended tris(aminophenyl)amine (TAPA) (0.50 g, 1.72 mmol) and dimethoxymethane (0.6 mL, 17.6 mmol) were stirred in DCM (10 mL), followed by dropwise addition of TFA (4.9 mL, 64.1 mmol). The reaction was left to stir for 16 hours, to yield a yellow solid. (0.5 g, 84% yield) BET: (CO_2, 273 K) = 380 m^2 g^{-1}; Total pore volume = 0.04 (at P/P_0 ~ 0.98); TGA: initial mass loss at 440 °C; FT-IR: ν max (cm^{-1}) 3662, 2988, 2902, 1982, 1655, 1605, 1485, 1407, 1230, 1066, 929, 824, 719, 530.

PIM-TAPA-(2TB)

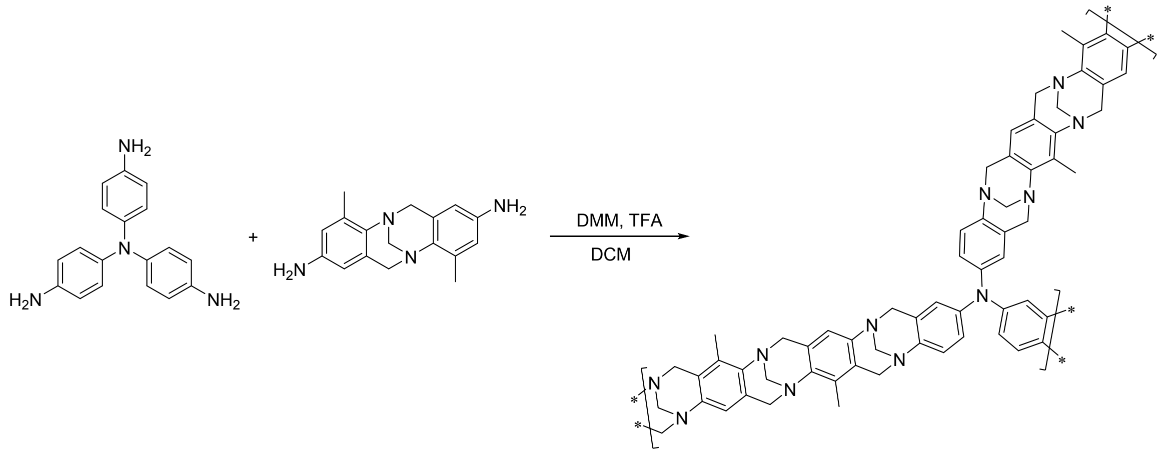

General procedure B was followed using tri(aminophenyl)amine (TAPA) (0.50 g, 1.72 mmol), 2TB (0.72 g, 2.58 mmol) and dimethoxymethane (1.22 mL, 13.77 mmol) were stirred in DCM (8 mL), followed by dropwise addition of TFA (5.93 mL, 77.4 mmol). The reaction was left to stir for 16 hours, to yield a pale-yellow solid. (1.20 g, 74%) BET: (CO_2, 273 K) = 270 m^2 g^{-1}; Total pore volume = 0.03 (at P/P_0 ~ 0.98); TGA: initial mass loss at 440 °C; FT-IR: ν max (cm^{-1}) 3663, 2924, 1669, 1606, 1505, 1200, 1066, 929, 826, 530.

PIM-TAPA-(Tol)-TB

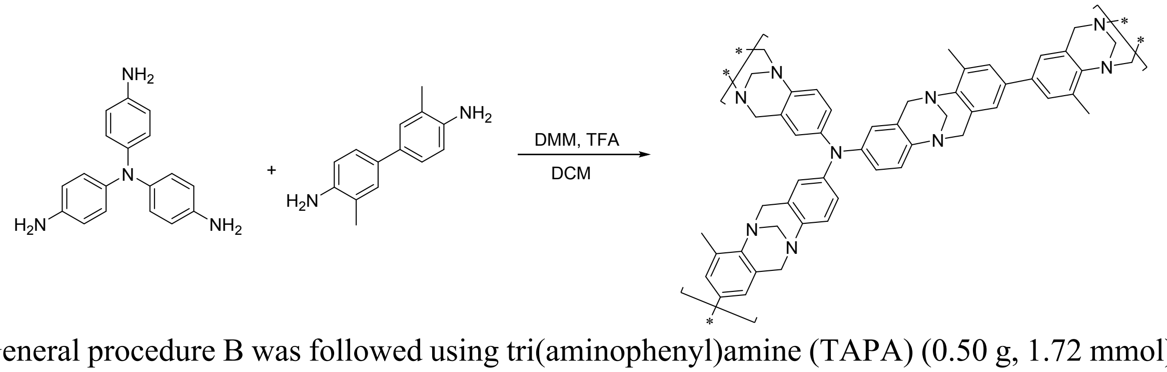

General procedure B was followed using tri(aminophenyl)amine (TAPA) (0.50 g, 1.72 mmol), tolidine (Tol) (0.55 g, 2.58 mmol) and dimethoxymethane (1.22 mL, 13.77 mmol) were stirred in DCM (6 mL), followed by dropwise addition of TFA (5.93 mL, 77.4 mmol). The reaction was left to stir for 16 hours, to yield a pale-yellow solid. (1.06 g, 78%) BET: (CO_2, 273 K) = 260 m^2 g^{-1}; Total pore volume = 0.01 (at P/P_0 ~ 0.98); TGA: initial mass loss at 420 °C; FT-IR: ν max (cm^{-1}) 3361, 1668, 1517, 1480, 1405, 1218, 804, 510.

3. Gas adsorption analysis

CO_2 isotherms

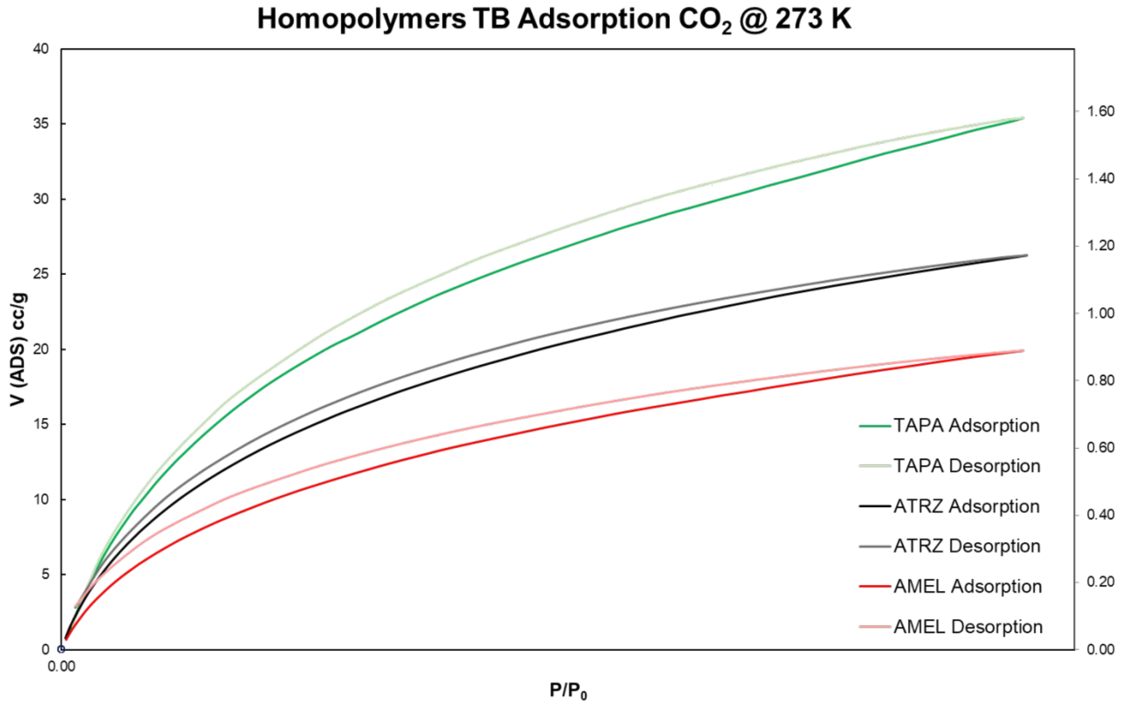

Figure S1. Adsorption of CO_2 at 273 K for the series of homo-polymers.

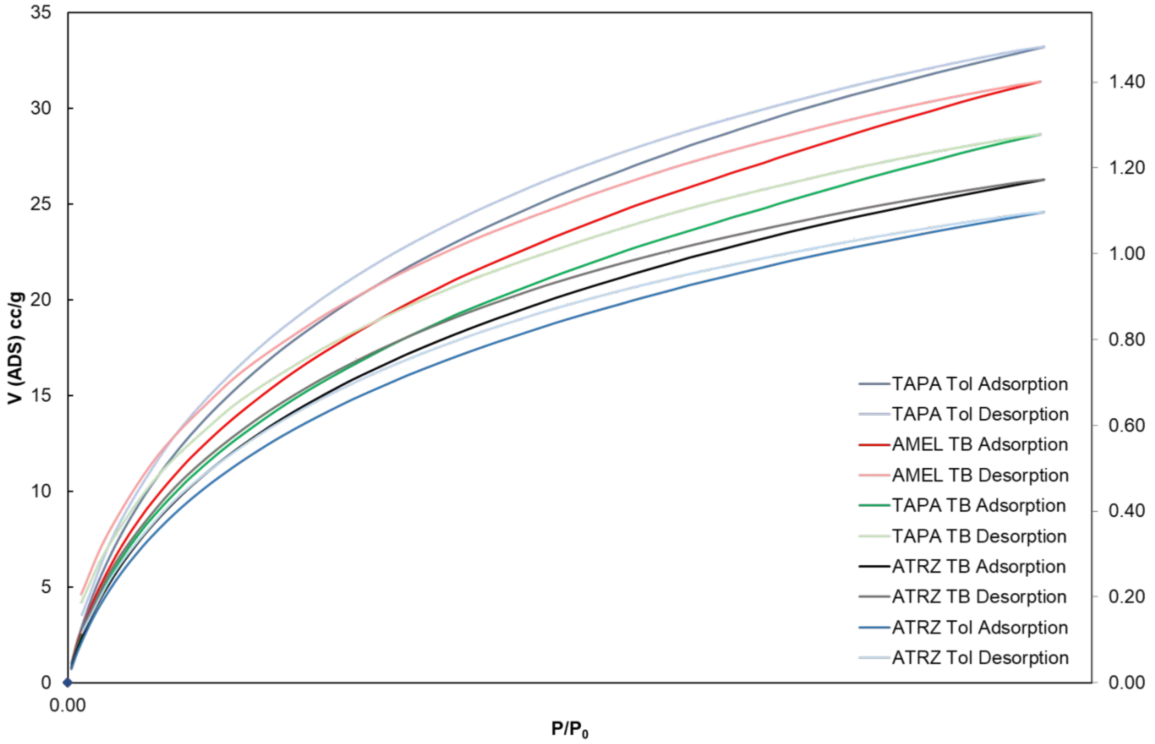

Figure S2. Adsorption of CO_2 at 273 K for the series of co-polymers.

Pore size distributions

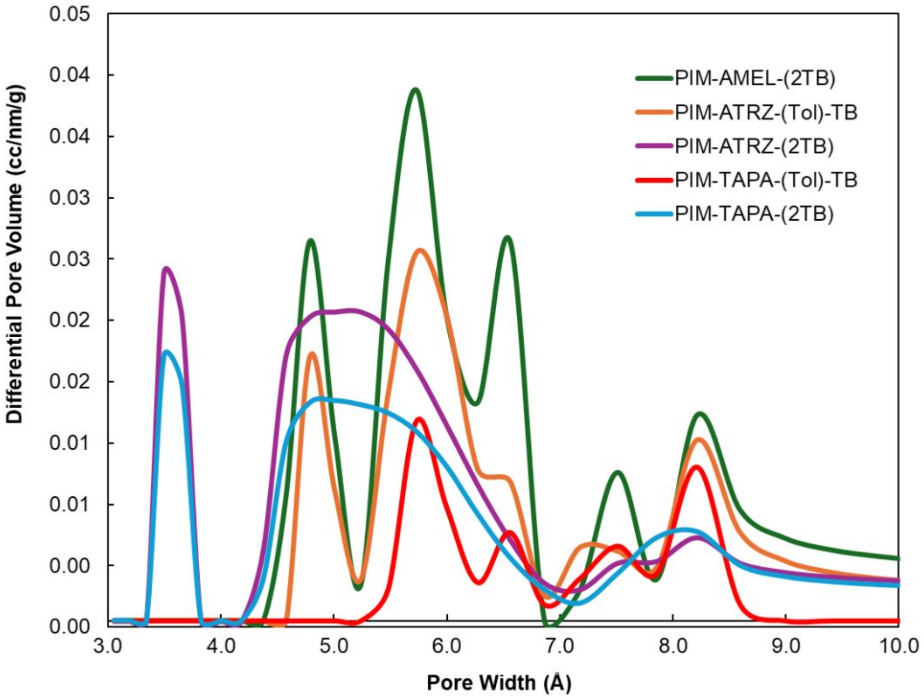

Figure S3. Pore size distribution of co-polymers calculated from CO_2 at 273 K via the NLDFT model.

4. General catalysis test:

Malononitrile (or other methylene species): benzaldehyde (1:3) solvent free:

A glass vial was charged with a mixture of benzaldehyde (15 mmol) and malononitrile (5 mmol), then the catalyst was added (1 mol%), and the reaction mixture was stirred at room temperature for 2 h. Fractions (10 microliters) were removed each 10 min and analysed by ^{1}H NMR.

Malononitrile (or other methylene species): benzaldehyde (1:3) solvent

A glass vial was charged with a mixture of benzaldehyde (15 mmol), malononitrile (5 mmol) and 2 mL of solvent (ethanol or DCM). Then, the catalyst (1 mol%) was added and the reaction mixture was stirred at room temperature for 2 h. Fractions (10 microliters) were removed each 10 min and analysed by ^{1}H NMR.

Malononitrile (or other methylene species): benzaldehyde (1:1) solvent

A glass vial was charged with a mixture of benzaldehyde (5 mmol), malononitrile (5 mmol) and 2 mL of solvent (ethanol or DCM). Then, the catalyst (1mol%) was added and the reaction mixture was stirred in at room temperature for 2 h. Fractions (10 microliters) were removed each 10 min and analysed by ^{1}H NMR.

Catalysis tables and figures

Table S1: Conversions in Knoevenagel reaction with 1:1 tBu-benzaldehyde: malononitrile in ethanol

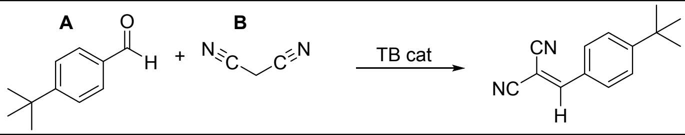

Entry	Catalyst	Conversion (%) at varying times (mins)						At completion		At 20 minutes	
		20	40	60	90	120	180	TON [a] (mol mol^{-1})	TOF [b] (h^{-1})	TON [a] (mol mol^{-1})	TOF [a] (h^{-1})
**1**	PIM-TAPB-TB[8]	9	17	26	33	38	55	37	12	6	18
**2**	PIM-TAPA-(2TB)	65	81	**96**				32	32	22	66
**3**	PIM-TAPA-TB	33	44	57	67	72	82	55	18	22	66
**4**	PIM-ATRZ-(2TB)	60	70	84	**100**			33	22	20	60
**5**	PIM-AMEL-TB	56	78	86	**96**			**64**	**43**	**37**	**113**
**6**	PIM-ATRZ-TB	14	17	22	28	36	49	33	11	9	28
**10**	PIM-AMEL-(2TB)	31	44	55	68	75	81	27	9	10	31
**11**	PIM-TAPB+A1-TB	43	57	65	74	78	**95**	48	16	22	66
**12**	PIM-TAPBext+A1-TB[8]	45	60	70	78	85	**95**	48	16	23	68

[a]Turnover number after 20 minutes and at maximum conversion time, calculated from no. of moles of malononitrile consumed versus no. of mole equivalents of TB catalyst. [b] Turnover frequency calculated from turnover number per hour.

Table S2: Results of the Knoevenagel reaction between benzaldehyde and varying methylene species (3:1) at 50 °C in ethanol

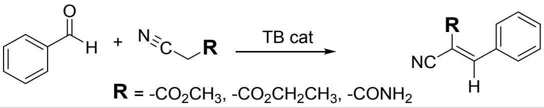

Methyl Species	*Catalyst (1 mol%)*	*% Conversion after x time*				At maximum conversion	
		2h	*4h*	*6h*	*24h*	TON [a] (mol mol^{-1})	TOF [b] (h^{-1})
Methyl Cyanoacetate	PIM-TAPA-TB	44	70	83	**100**	**67**	**3**
	PIM-TAPA-(2TB)	52	75	86	**100**	33	1.4
	PIM-AMEL-TB	54	77	88	**100**	**67**	**3**
	PIM-AMEL-(2TB)	53	83	94	**100**	33	1.4
Ethyl Cyanoacetate	PIM-TAPA-TB	29	53	65	94	63	2.6
	PIM-TAPA-(2TB)	32	44	60	90	30	1.25
	PIM-AMEL-TB	32	58	70	**97**	**65**	**2.7**
	PIM-AMEL-(2TB)	23	44	61	**98**	33	1.4
Cyanoacetamide	PIM-TAPA-TB	23	40	50	78	52	2.2
	PIM-TAPA-(2TB)	32	44	57	74	25	1
	PIM-AMEL-TB	40	59	71	**89**	**59**	**2.5**
	PIM-AMEL-(2TB)	27	52	63	**82**	27	1.1

[a]Turnover number at maximum conversion, calculated from no. of moles of methylene species consumed versus no. of mole equivalents of TB catalyst. [b] Turnover frequency calculated from turnover number per hour.

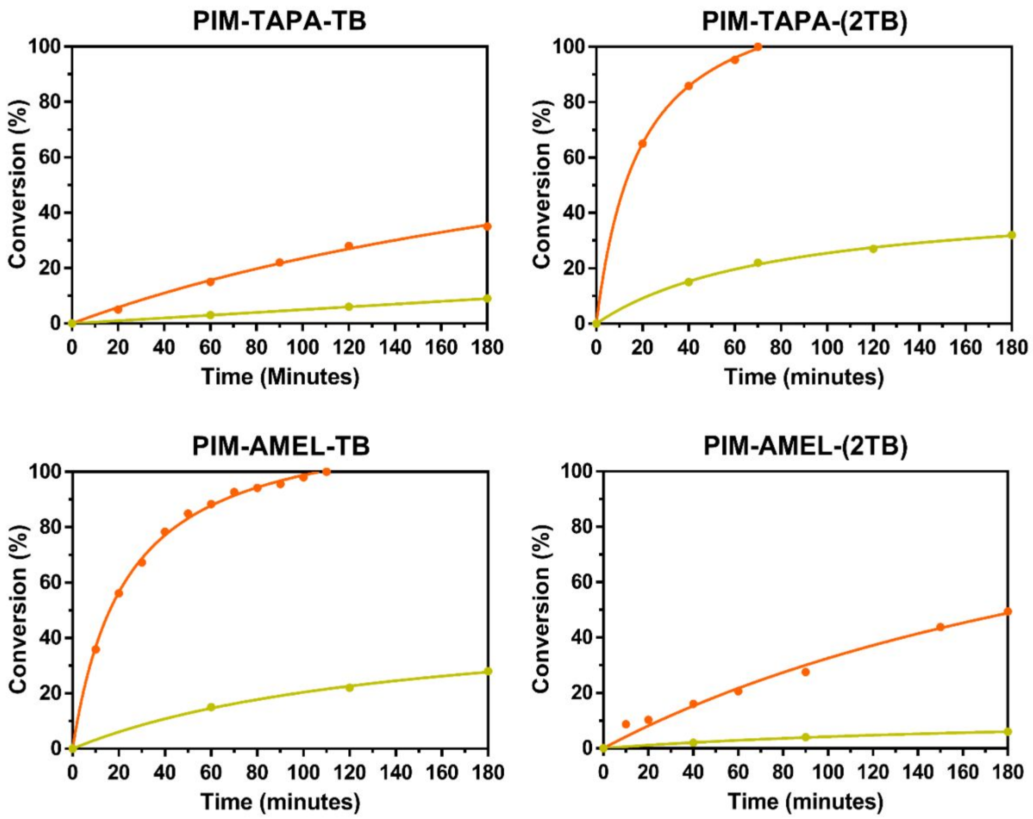

Figure S4: Performance of polymers with *t*Bu benzaldehyde in solvent-free and ethanol conditions. (Fitted with Prism as Hyperbola or Sigmoidal 4PL (both least squares, 99%).

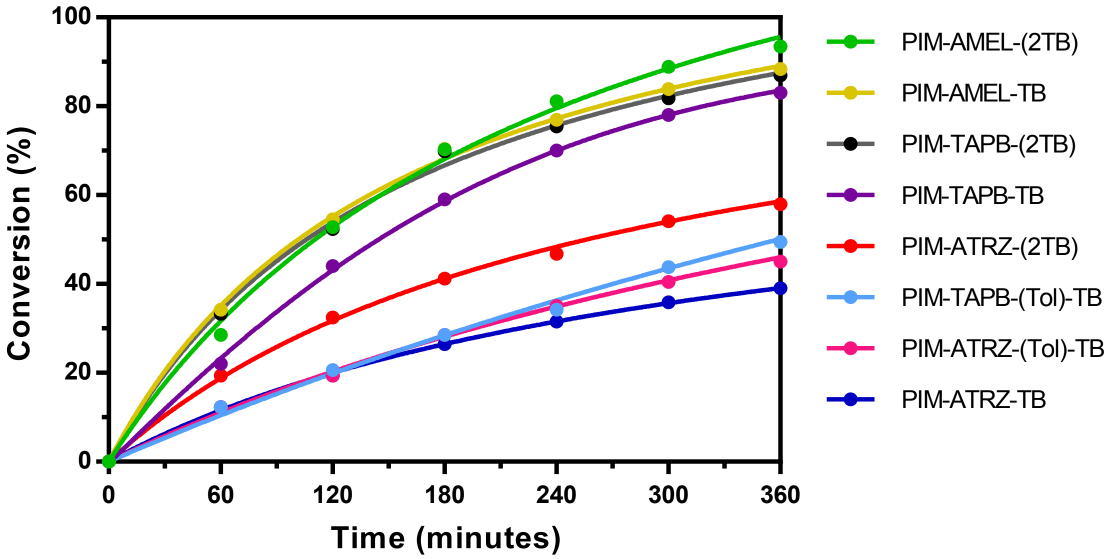

Figure S5. Performance of polymers in the reaction between 3:1 benzaldehyde and methyl cyanoacetate at 50 °C in ethanol. (Fitted with Prism as Hyperbola or Sigmoidal 4PL (both least squares, 99%).

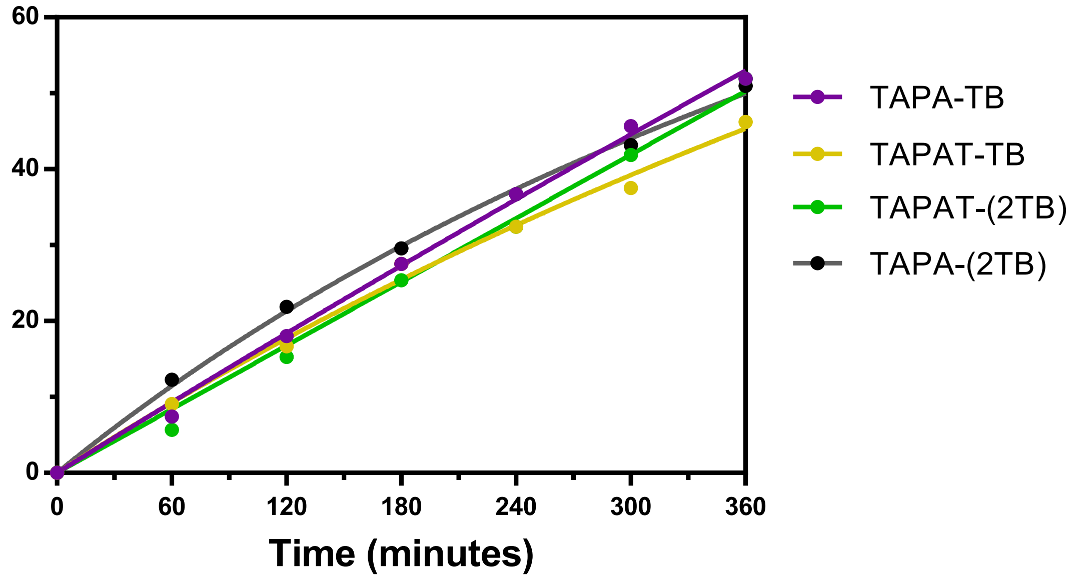

Figure S6: Performance of polymers in the reaction 3:1 benzaldehyde and methyl cyanoacetate at 25 °C. (Fitted with Prism as Hyperbola or Sigmoidal 4PL (both least squares, 99%).

5. Reaction kinetics

Under normal circumstances, if there are no rate-limiting intermediate steps or other phenomena, like diffusion limitations of the reagents or products to or from the catalytic site, and if both reactants are involved in the rate-limiting step, the Knoevenagel condensation reaction is expected to be first order in the nitrile A and the aldehyde B:

$$\frac{dC_A}{dt} = \frac{dC_B}{dt} = -kC_AC_B \qquad \text{SEq. 1}$$

Where C_A and C_B are the concentrations of both reagents and *k* is the reaction rate constant. Considering the stoichiometric reaction, we can substitute C_B at every moment:

$$C_B = C_{B,0} - (C_{A,0} - C_A) \qquad \text{SEq. 2}$$

Thus, when both reactants are involved in the rate-limiting step, integration of SEq. 1 from $t = 0$, where $C_A = C_{A,0}$ and $C_B = C_{B,0}$, gives:

$$\frac{1}{C_{B,0} - C_{A,0}} \cdot \ln\left(\frac{C_B \cdot C_{A,0}}{C_A \cdot C_{B,0}}\right) = -k\,t \qquad \text{SEq. 3}$$

and for C_A:

$$C_A = \frac{C_{B,0} - C_{A,0}}{\left(\frac{C_{B,0}}{C_{A,0}} e^{(C_{B,0} - C_{A,0})} \cdot k\,t - 1\right)} \qquad \text{SEq. 4}$$

In the not unlikely case that the rate limiting step involves only one of the reagents, we would instead have a simple first order reaction:

$$\frac{dC_A}{dt} = -kC_A \text{ or } \frac{dC_B}{dt} = -kC_B \qquad \text{SEq. 5}$$

which describes a simple exponential decay function in the concentration of either A or B:

$$C_A = C_{A,0}\, e^{-k\,t} \; or \; C_B = C_{B,0}\, e^{-k\,t} \qquad \text{SEq. 6}$$

SEq. 4 has no solution if both reagents have the same concentration, *i.e.* if $C_{A,0} = C_{B,0}$ and $C_A = C_B$. However, in this case SEq. 1 can be written as:

$$\frac{dC_A}{dt} = -k\,C_A^2 \quad \text{or} \quad \frac{dC_B}{dt} = -k\,C_B^2 \qquad \text{SEq. 7}$$

Thus, SEq. 1 transforms into a simple second order reaction in either A or B. Considering again the stoichiometric reaction, substitution of C_B by SEq. 2, and integration of SEq. 7 gives the following concentration profiles:

$$\frac{1}{C_A} - \frac{1}{C_{A,0}} = k\,t \;\; or \;\; \frac{1}{C_B} - \frac{1}{C_{B,0}} = k\,t \qquad \text{SEq. 8}$$

and thus:

$$C_A = \frac{1}{1 + k\,t\,C_{A,0}} \quad \text{or} \quad C_B = \frac{1}{1 + k\,t\,C_{B,0}} \qquad \text{SEq. 9}$$

A more complex situation might arise when the reaction still involves both reagents, but diffusion limitations reduce their concentration near the active site, or affinity of the porous catalyst for the reagents increases their concentration near the catalytically active site. If we assume that the deviation of the reagent concentrations near the active site, $C_{A,cat}$ and $C_{B,cat}$, from that in the bulk is defined by a power function:

$$C_{A,cat} = C_A^n \qquad \text{SEq. 10}$$

and

$$C_{B,cat} = C_B^m \qquad \text{SEq. 11}$$

Where n, $m < 1$ if the concentration in the catalyst is lower than in the bulk due to transport limitations and n, $m > 1$ if the concentration in the catalyst is higher than that in the bulk liquid. Thus, SEq. 1 changes into:

$$\frac{dC_A}{dt} = \frac{dC_B}{dt} = -kC_A^n C_B^m \qquad \text{SEq. 12}$$

The integral of this equation cannot be solved analytically to give a similar analytical function for the concentration profile as a function of time like in SEq. 4, but the profile can be obtained

by numerical integration of SEq. 12 and a least squares estimation of the parameters k, n and m.

$$C_A(t) = \sum_{t=0}^{t=t} \frac{\Delta C_A}{\Delta t} \Delta t = \sum_{\substack{t=0,\\ C_{A,0},\, C_{B,o}}}^{\substack{t=t,\\ C_{A,t},\, C_{B,t}}} \left(-kC_{\mathrm{A}}^{n} C_{\mathrm{B}}^{m}\right) \Delta t \qquad \text{SEq. 13}$$

For simplicity, in this work we consider the situation where $n = m$. **Figure 8** in the manuscript shows an example of the fit of the reaction of equimolar amounts of t-Bu-benzaldehyde and malononitrile in ethanol, catalyzed by PIM-TAPA-(2TB) and PIM-AMEL-(2TB), which show radically different reaction kinetics. While the reaction with PIM-TAPA-(2TB) proceeds via a first order reaction in malononitrile, the reaction with PIM-AMEL-(2TB) is much better described by a second order kinetics. It must be noted that in the presence of equimolar amounts of both reagents, it is not possible to establish with a single reaction which of the two reactants is involved in the rate-limiting step but it seems more likely that it is the bulkier aldehyde than malononitrile.

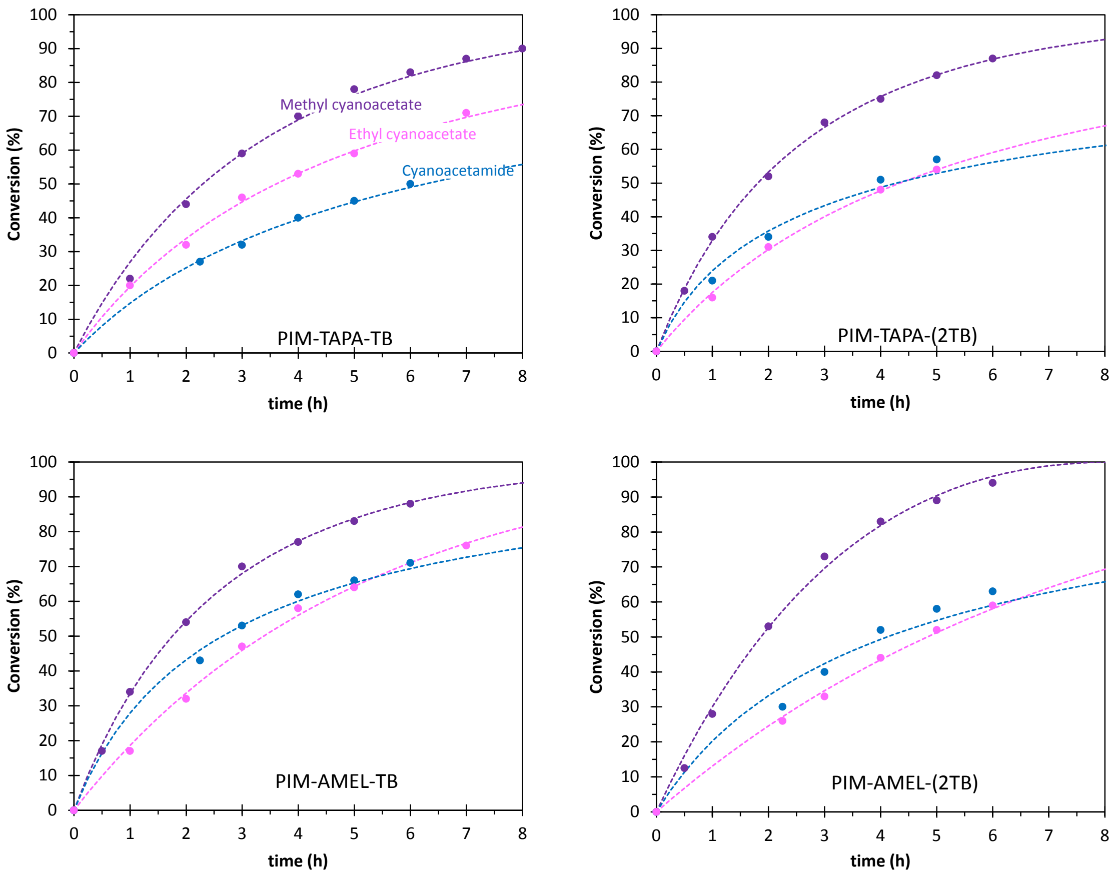

Figure S7. Zoom of the early reaction stage and curve fit of the data in **Figure 4** with the model $\frac{dC_A}{dt} = \frac{dC_B}{dt} = -kC_{\mathrm{A}}^{n} C_{\mathrm{B}}^{m}$

6. ¹³C Solid State NMR

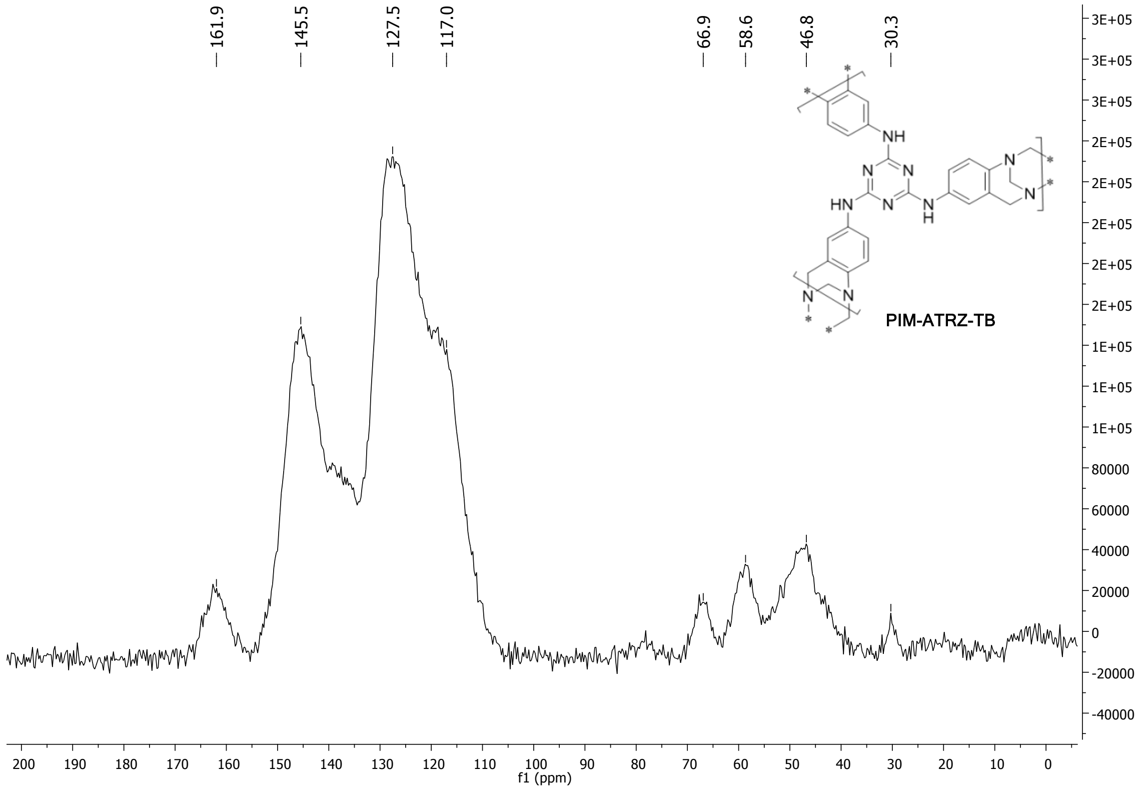

Figure S8. ^{13}C SS NMR of PIM-ATRZ-TB.

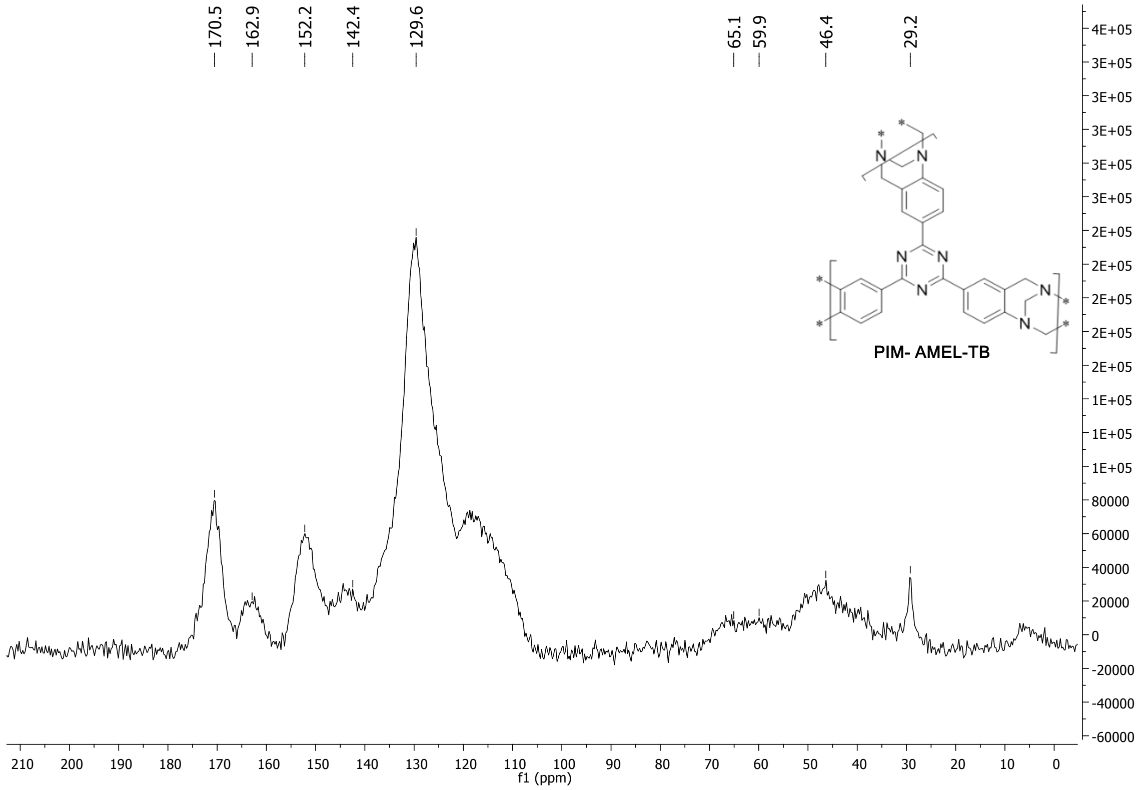

Figure S9. ^{13}C SS NMR of PIM-AMEL-TB.

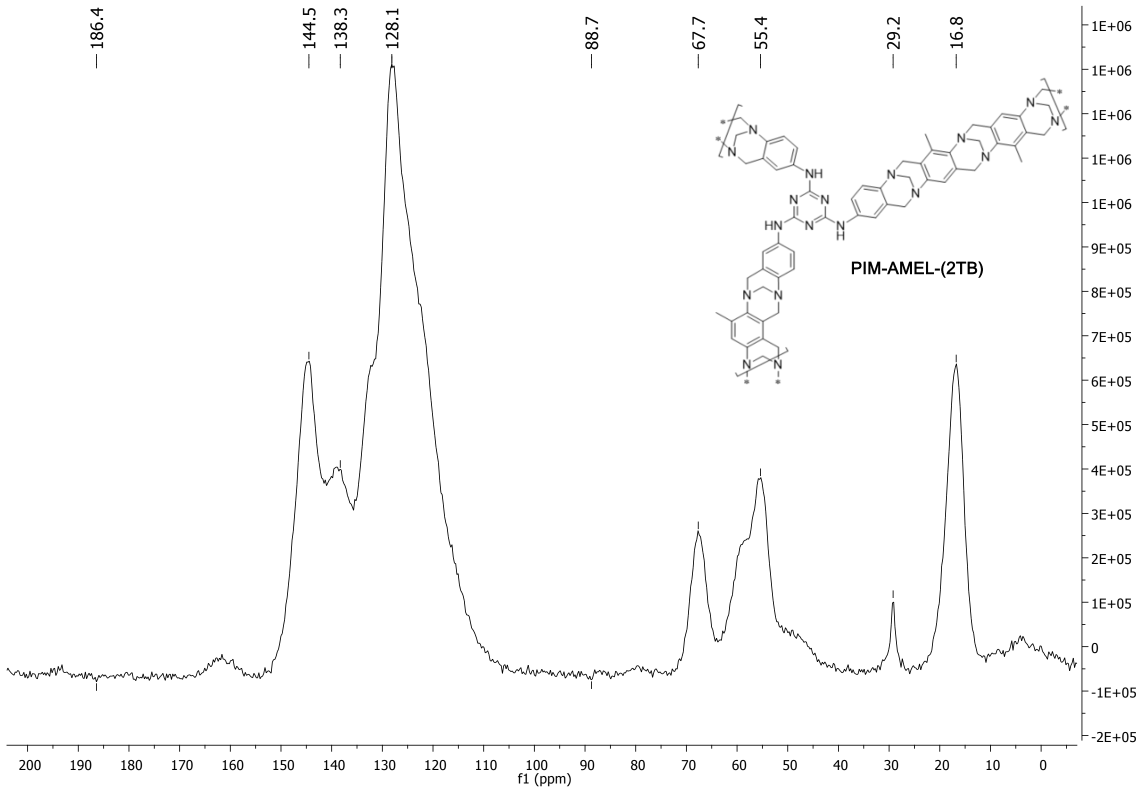

Figure S10. ^{13}C SS NMR of PIM-AMEL-(2TB).

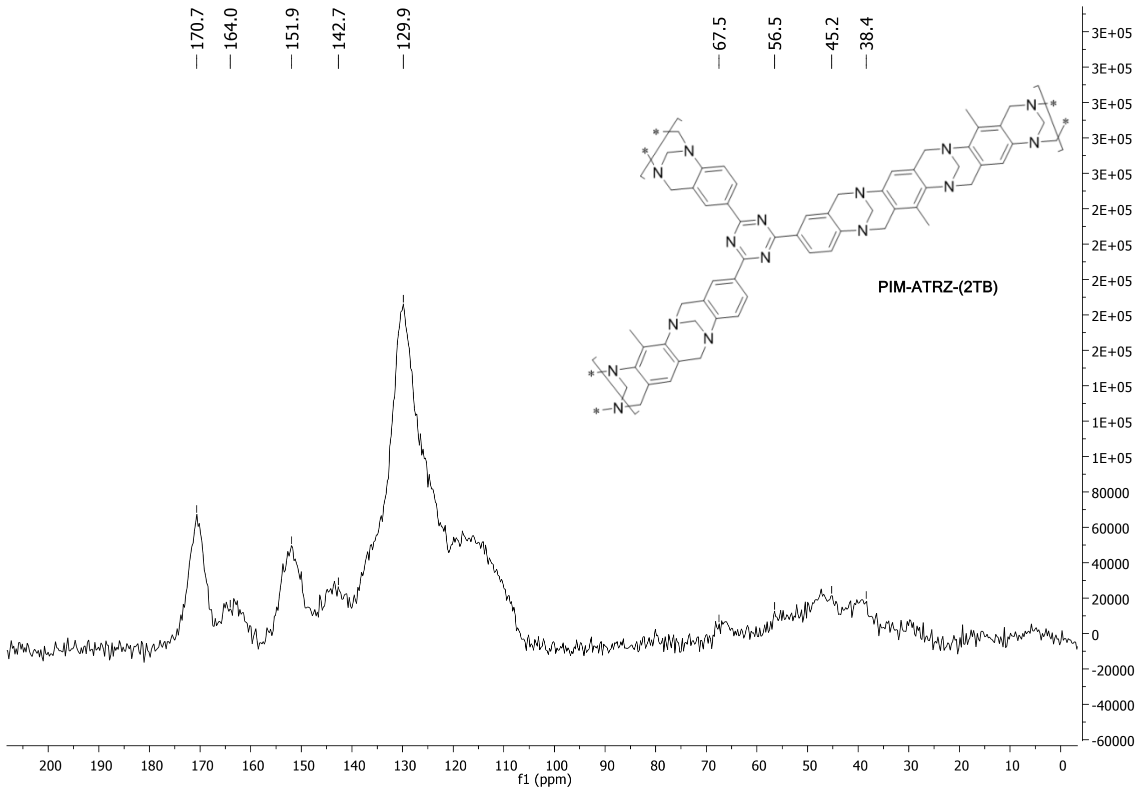

Figure S11. ^{13}C SS NMR of PIM-ATRZ-(2TB).

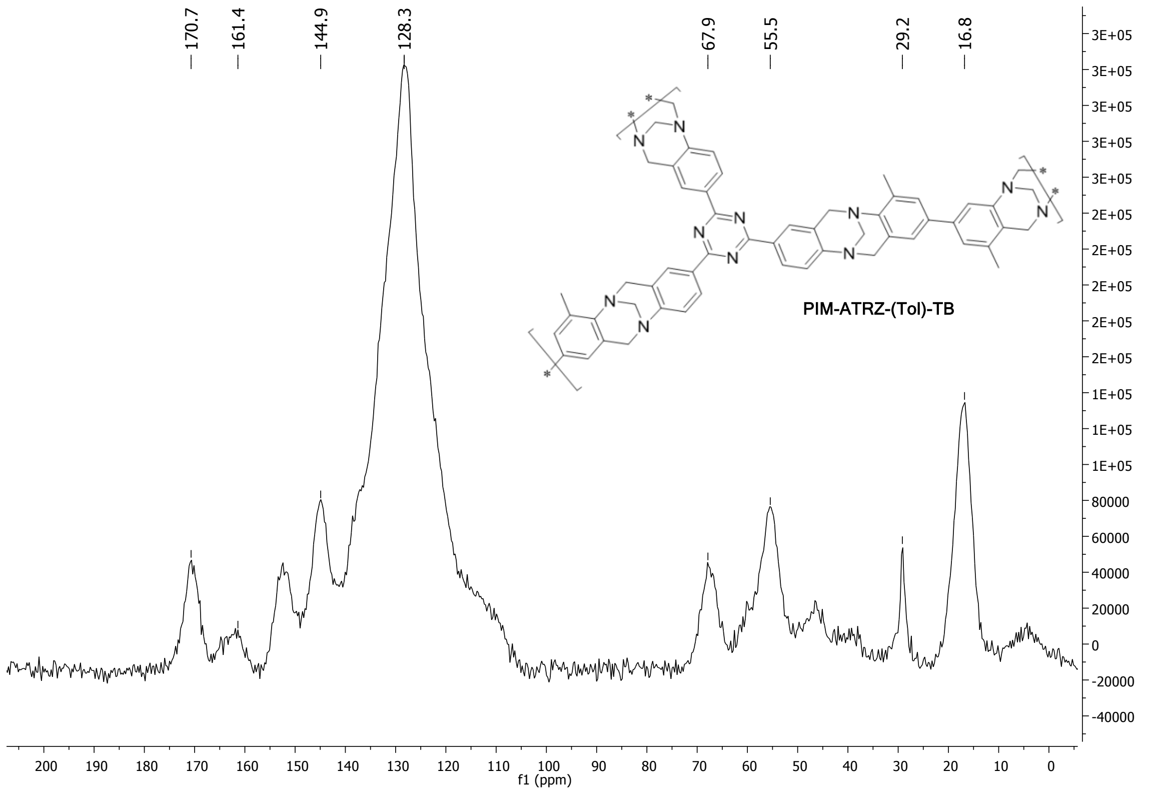

Figure S12. ^{13}C SS NMR of PIM-ATRZ-(Tol)-TB.

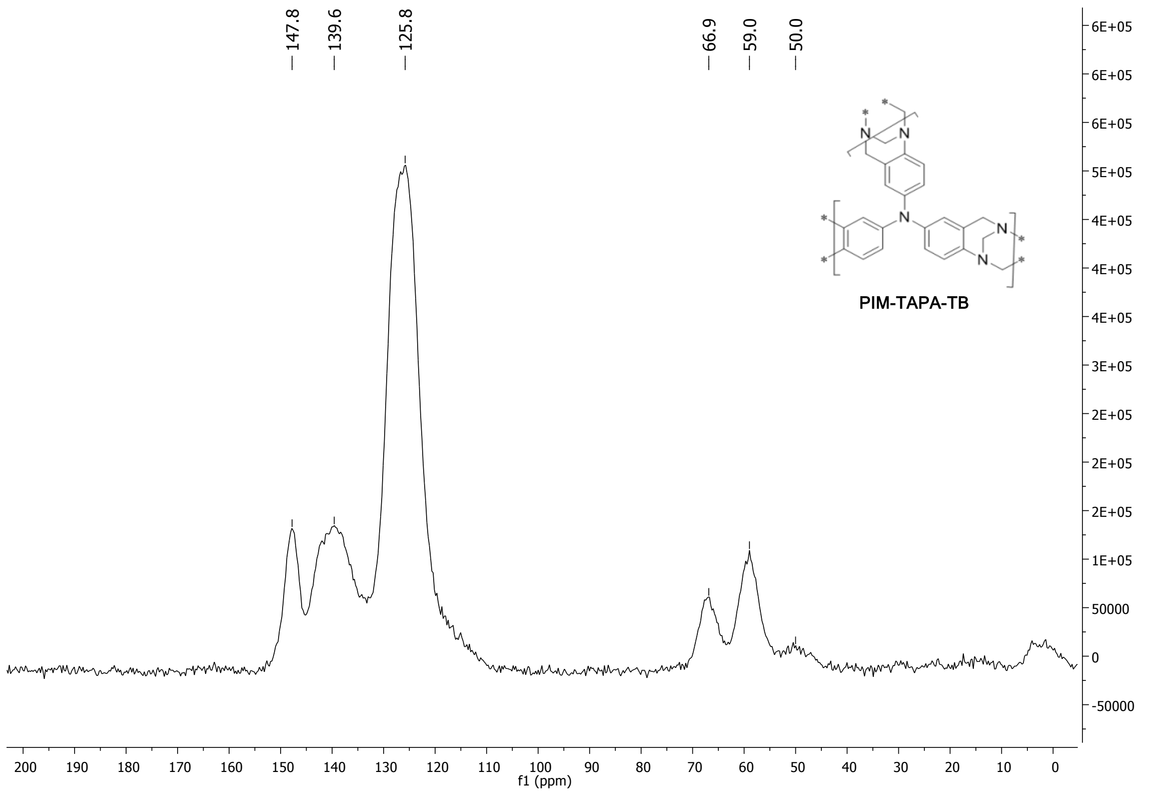

Figure S13. ^{13}C SS NMR of PIM-TAPA-TB.

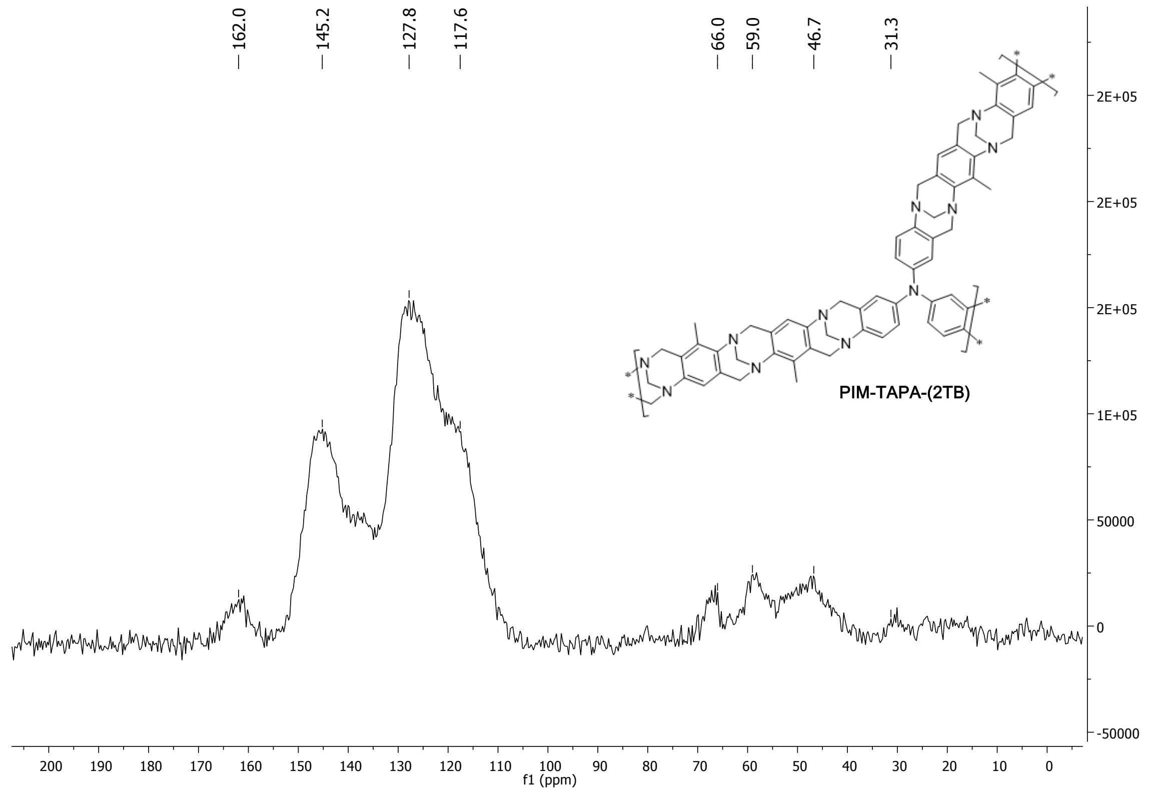

Figure S14. ^{13}C SS NMR of PIM-TAPA-(2TB).

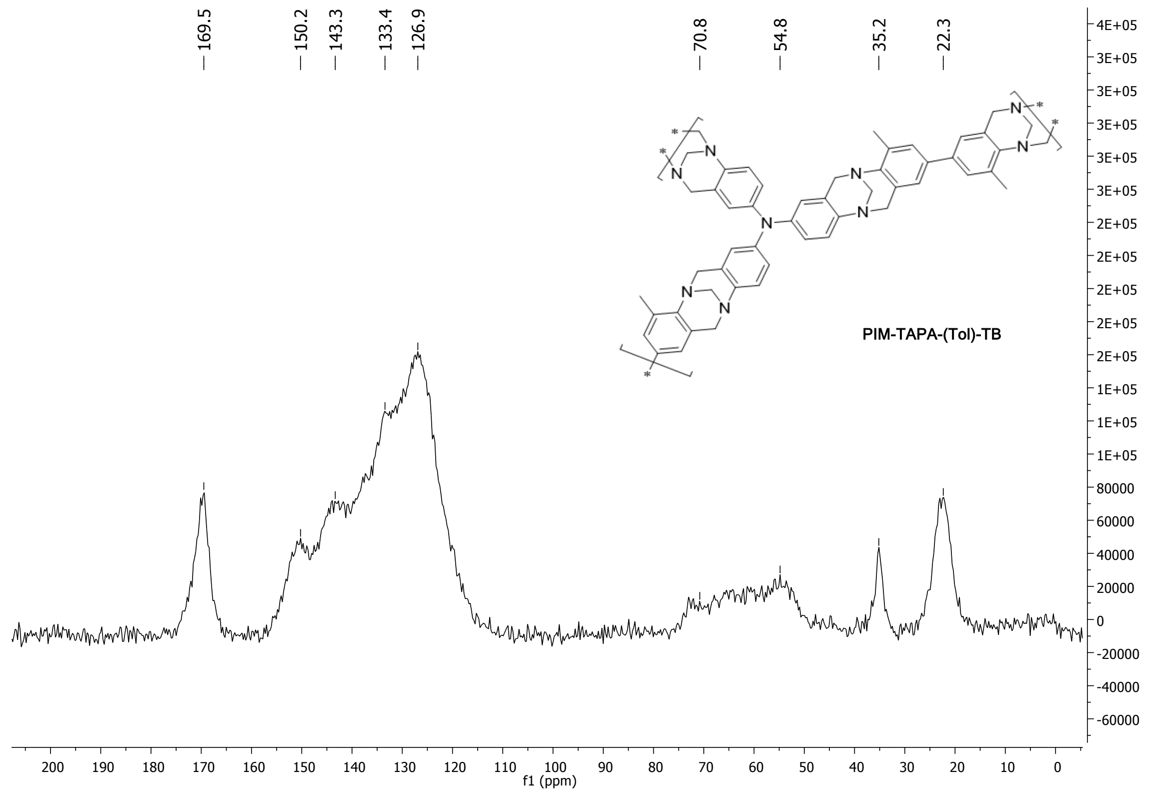

Figure S15. ^{13}C SS NMR of PIM-TAPA-(Tol)-TB.

7. Computational Studies

Computational details

Quantum Mechanical calculations were performed in the frame of Density Functional Theory using NWChem 7.0.2 software.[9] The geometries were optimized allowing the relaxation of the entire structures, using the CAM-B3LYP functional.[10] This hybrid exchange–correlation functional was selected since it combines the good ability of the well-known B3LYP functional to reproduce the atomization energies, and better performs on the charge transfer excitations. 6-31G** basis sets were used for all the calculations, the energy convergence threshold was set to 10^{-6} a.u., and the convergence criteria for the geometry optimization were the maximum and root-mean-square of total energy gradients equal to 4.5×10^{-4} and 3.0×10^{-4} a.u., respectively, and the maximum and root-mean-square of the Cartesian displacement vectors equal to 1.8×10^{-3} and 1.2×10^{-3} a.u, respectively. Partial electrical charges have been calculated by the ESP method embedded in the same NWChem version.

Chemical structures of TAPBext-PIM, PIM-TAPA, PIM-AMEL-TB

TAPBext-PIM

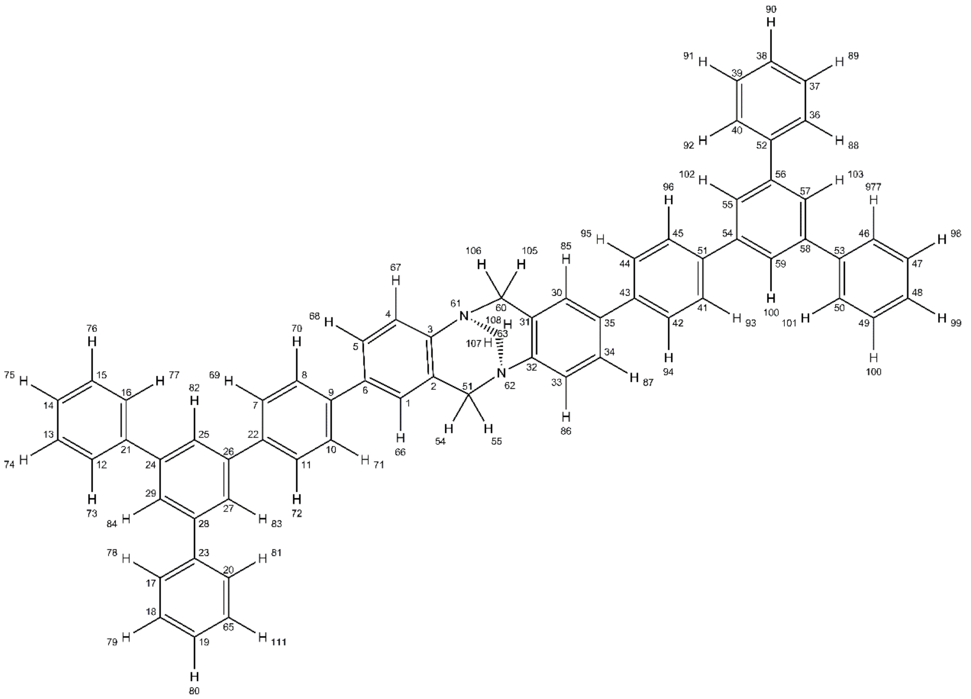

PIM-TAPA

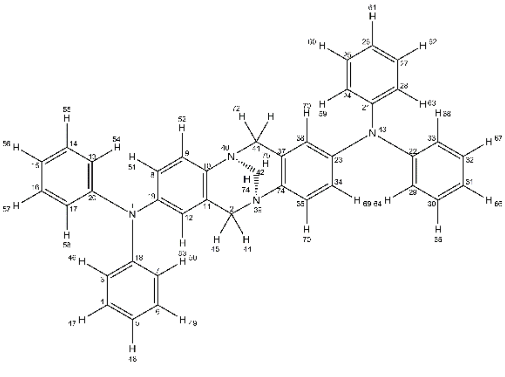

PIM-AMEL-TB

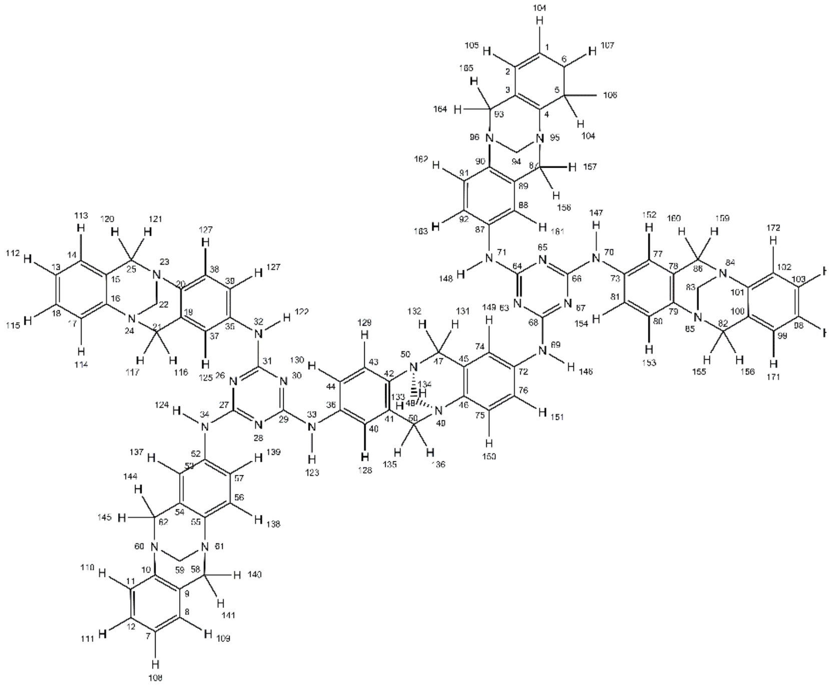

Figure S16. Chemical structures of the TAPBext-PIM, PIM-TAPA, and PIM-AMEL-TB that were studied in this work.

Table S3: Calculated HOMO and LUMO energies and energy gap (ΔE_{gap}) of TAPBext-PIM, PIM-TAPA, and PIM-AMEL-TB.

	**TAPBext-PIM**	**PIM-TAPA**	**PIM-AMEL-TB**
**E_{HOMO} (eV)**	-6.78	-6.10	-6.50
**E_{LUMO} (eV)**	0.16	0.80	0.73
**ΔE_{gap} (eV)**	6.94	6.90	7.23

Table S4: Optimized geometry of the TAPBext-PIM including the ESP calculation results.

Atom		Coordinates			Charge
Number	Element	x	y	z	ESP
1	C	0.344896	-0.284458	-0.037704	-0.185702
2	C	0.43037	-0.175289	-0.025236	-0.305373
3	C	0.461884	-0.127511	0.102524	0.501807
4	C	0.40425	-0.188148	0.214179	-0.274544
5	C	0.317777	-0.295148	0.199876	-0.169081
6	C	0.28717	-0.346192	0.073118	0.106154
7	C	0.115542	-0.678148	0.132859	-0.187441
8	C	0.200115	-0.569258	0.147378	-0.116734
9	C	0.196358	-0.462343	0.057435	0.073374

10	C	0.104385	-0.468907	-0.047597	-0.147408
11	C	0.01991	-0.577891	-0.062228	-0.141383
12	C	-0.404595	-1.077901	0.228263	-0.119644
13	C	-0.461913	-1.13699	0.340083	-0.127885
14	C	-0.387775	-1.152757	0.456607	-0.072988
15	C	-0.255882	-1.109009	0.460293	-0.088331
16	C	-0.198628	-1.050118	0.348325	-0.135882
17	C	-0.340415	-1.057771	-0.298899	-0.252411
18	C	-0.368302	-1.10841	-0.425148	0.012829
19	C	-0.267841	-1.116913	-0.520843	-0.156563
20	C	-0.11153	-1.0239	-0.362833	-0.177375
21	C	-0.272042	-1.033609	0.23054	0.063257
22	C	0.023766	-0.684762	0.027711	0.10895
23	C	-0.211624	-1.014773	-0.265702	0.174667
24	C	-0.210849	-0.970636	0.110841	0.094182
25	C	-0.124646	-0.861707	0.123882	-0.184283
26	C	-0.066491	-0.801481	0.012228	0.034709
27	C	-0.096243	-0.852159	-0.114325	-0.15326
28	C	-0.182123	-0.960898	-0.130533	0.020992
29	C	-0.238542	-1.019195	-0.017007	-0.195995
30	C	0.344597	0.28428	0.040619	-0.226341
31	C	0.430199	0.175153	0.028784	-0.302308
32	C	0.462717	0.1274	-0.098738	0.469184
33	C	0.405976	0.188098	-0.210825	-0.242504
34	C	0.319431	0.295123	-0.19716	-0.197531
35	C	0.28781	0.3461	-0.070634	0.140994
36	C	-0.341992	1.058113	0.296356	-0.250391
37	C	-0.370654	1.109134	0.422273	-0.038045
38	C	-0.270703	1.118225	0.518452	-0.119303
39	C	-0.141938	1.075906	0.487598	-0.08291
40	C	-0.113358	1.025007	0.361586	-0.179302
41	C	0.116506	0.677912	-0.131892	-0.121848
42	C	0.201211	0.569044	-0.145722	-0.176314
43	C	0.196918	0.462258	-0.055654	0.087023
44	C	0.104297	0.468932	0.048797	-0.156908
45	C	0.019683	0.577895	0.062746	-0.143915
46	C	-0.40319	1.077257	-0.23104	-0.147163
47	C	-0.459982	1.136135	-0.343235	-0.112773
48	C	-0.38521	1.15194	-0.459345	-0.049423
49	C	-0.253213	1.108445	-0.462238	-0.132465
50	C	-0.196493	1.049723	-0.349907	-0.109606
51	C	0.024063	0.68463	-0.027338	0.052754
52	C	-0.212927	1.015321	0.26398	0.191598

53	C	-0.270563	1.03316	-0.232541	0.091995
54	C	-0.066297	0.80136	-0.012648	0.106266
55	C	-0.096706	0.852331	0.113633	-0.209064
56	C	-0.182635	0.961125	0.129123	0.009324
57	C	-0.238376	1.019211	0.015162	-0.157667
58	C	-0.21002	0.970364	-0.112422	0.009265
59	C	-0.123834	0.861339	-0.124761	-0.143304
60	C	0.488256	0.108456	0.152205	0.742125
61	N	0.553381	-0.019196	0.121037	-0.866452
62	N	0.554318	0.019042	-0.116533	-0.843449
63	C	0.635903	-0.000126	0.00257	0.701582
64	C	0.489398	-0.108589	-0.148201	0.70673
65	C	-0.139341	-1.074396	-0.489175	-0.062289
66	H	0.323252	-0.322898	-0.137062	0.095218
67	H	0.42979	-0.150529	0.312732	0.14392
68	H	0.272631	-0.339704	0.287895	0.120068
69	H	0.122961	-0.761042	0.202497	0.117246
70	H	0.272608	-0.568475	0.228121	0.101575
71	H	0.096685	-0.385893	-0.117062	0.107585
72	H	-0.052612	-0.578661	-0.142949	0.099247
73	H	-0.463917	-1.063712	0.138513	0.10343
74	H	-0.565206	-1.170096	0.336389	0.099829
75	H	-0.43253	-1.198868	0.54404	0.088689
76	H	-0.196906	-1.121532	0.550536	0.082956
77	H	-0.09488	-1.018437	0.351252	0.10433
78	H	-0.419913	-1.049322	-0.225517	0.129016
79	H	-0.469137	-1.140654	-0.449061	0.06781
80	H	-0.289571	-1.156465	-0.619521	0.100052
81	H	-0.010389	-0.993262	-0.338177	0.099586
82	H	-0.106113	-0.820121	0.222334	0.111249
83	H	-0.051675	-0.806108	-0.201844	0.111944
84	H	-0.3015	-1.106826	-0.02806	0.114036
85	H	0.32206	0.322575	0.139833	0.114107
86	H	0.432241	0.150535	-0.309206	0.137687
87	H	0.275	0.339755	-0.285506	0.12732
88	H	-0.421082	1.049199	0.222589	0.135591
89	H	-0.47169	1.141213	0.445547	0.086079
90	H	-0.293032	1.15811	0.616863	0.091498
91	H	-0.063008	1.083351	0.561717	0.097752
92	H	-0.012024	0.994505	0.337555	0.105689
93	H	0.124302	0.760717	-0.2016	0.098712
94	H	0.274199	0.568209	-0.226017	0.116278
95	H	0.096229	0.38601	0.11833	0.110805

96	H	-0.053349	0.578731	0.143006	0.101513
97	H	-0.462959	1.063133	-0.141579	0.110464
98	H	-0.563354	1.169055	-0.340153	0.095984
99	H	-0.429554	1.19789	-0.547071	0.07677
100	H	-0.193747	1.121034	-0.552149	0.100463
101	H	-0.092674	1.018225	-0.352231	0.095257
102	H	-0.052614	0.806455	0.201489	0.13121
103	H	-0.301328	1.10691	0.025672	0.11037
104	H	-0.104847	0.819488	-0.223019	0.101383
105	H	0.562219	0.174254	0.199464	-0.096328
106	H	0.409603	0.091085	0.226018	-0.091908
107	H	0.699718	0.087223	0.017955	-0.056056
108	H	0.699661	-0.087597	-0.012337	-0.056899
109	H	0.563728	-0.174405	-0.194833	-0.088209
110	H	0.411342	-0.091183	-0.222643	-0.082245
111	H	-0.060007	-1.081381	-0.562906	0.092727

Table S5: Optimized geometry of the PIM-TAPA including the ESP calculation results.

Atom		Coordinates			Charge
Number	Element	x	y	z	ESP
1	N	0.039394	-0.460954	0.015452	-0.443319
2	C	-0.292406	-0.091144	0.017033	0.638899
3	C	-0.002555	-0.610822	0.203814	-0.271068
4	C	-0.081599	-0.70589	0.266871	-0.020816
5	C	-0.201203	-0.747782	0.209657	-0.240634
6	C	-0.240584	-0.693773	0.087746	-0.027584
7	C	-0.161346	-0.599931	0.023074	-0.278025
8	C	0.018503	-0.315197	-0.180094	-0.311636
9	C	-0.042846	-0.209457	-0.244797	-0.178268
10	C	-0.145249	-0.13718	-0.183409	0.384268
11	C	-0.184445	-0.171351	-0.053433	-0.235873
12	C	-0.122463	-0.278144	0.010868	-0.290258
13	C	0.262126	-0.361821	0.027334	-0.180791
14	C	0.400285	-0.374995	0.024889	-0.123652
15	C	0.459276	-0.500117	0.011004	-0.124209
16	C	0.378053	-0.612281	-0.001527	-0.090878
17	C	0.239828	-0.59993	-0.001022	-0.169549
18	C	-0.041349	-0.557225	0.080664	0.465227
19	C	-0.02154	-0.351344	-0.051057	0.450993
20	C	0.18026	-0.474345	0.014114	0.294861
21	C	0.168365	0.450343	-0.01149	0.341275

22	C	-0.014689	0.581008	0.087085	0.306333
23	C	-0.055903	0.352727	0.007107	0.507054
24	C	0.239032	0.331878	0.010998	-0.123091
25	C	0.37159	0.320771	-0.028592	-0.166148
26	C	0.436477	0.427631	-0.089454	-0.104394
27	C	0.366624	0.545852	-0.11127	-0.109618
28	C	0.233484	0.557246	-0.073683	-0.207541
29	C	-0.140846	0.631473	0.05427	-0.158058
30	C	-0.187336	0.747986	0.113707	-0.116535
31	C	-0.108689	0.817091	0.205178	-0.111011
32	C	0.017151	0.767509	0.237427	-0.099419
33	C	0.063741	0.650105	0.179923	-0.178765
34	C	-0.147248	0.315545	0.106362	-0.326505
35	C	-0.23507	0.211	0.084394	-0.192918
36	C	-0.232097	0.138265	-0.034662	0.38238
37	C	-0.138315	0.172209	-0.132902	-0.219881
38	C	-0.053364	0.280474	-0.111935	-0.344255
39	N	-0.326073	0.032238	-0.054661	-0.767674
40	N	-0.209468	-0.031449	-0.255296	-0.771806
41	C	-0.130838	0.092145	-0.261739	0.647412
42	C	-0.338745	0.000332	-0.196166	0.46888
43	N	0.032739	0.461528	0.027736	-0.458916
44	H	-0.260277	-0.065737	0.118321	-0.063648
45	H	-0.383578	-0.151354	0.026851	-0.068989
46	H	0.090005	-0.577589	0.249426	0.114691
47	H	-0.049807	-0.746501	0.362375	0.088886
48	H	-0.263016	-0.821674	0.259499	0.130905
49	H	-0.33333	-0.725992	0.041457	0.098218
50	H	-0.191849	-0.559103	-0.072606	0.103871
51	H	0.096656	-0.371805	-0.229512	0.138713
52	H	-0.014429	-0.183514	-0.346278	0.136662
53	H	-0.152336	-0.304512	0.11192	0.098862
54	H	0.216596	-0.264197	0.039548	0.095405
55	H	0.462273	-0.286509	0.03538	0.117841
56	H	0.567278	-0.510126	0.009906	0.097134
57	H	0.422504	-0.710607	-0.013255	0.099495
58	H	0.177051	-0.687603	-0.012137	0.086143
59	H	0.18916	0.248729	0.059492	0.070771
60	H	0.424892	0.228001	-0.010327	0.131545
61	H	0.540307	0.418846	-0.119583	0.094642
62	H	0.415622	0.629932	-0.159345	0.109569
63	H	0.17908	0.649193	-0.0921	0.107571
64	H	-0.20214	0.578666	-0.017882	0.092704

65	H	-0.285571	0.785675	0.08704	0.101793
66	H	-0.145045	0.908512	0.250839	0.093204
67	H	0.079479	0.819834	0.309249	0.099028
68	H	0.161369	0.611168	0.206474	0.098231
69	H	-0.150104	0.370696	0.199644	0.138649
70	H	-0.308411	0.184325	0.15982	0.143111
71	H	0.018125	0.308016	-0.188919	0.118291
72	H	-0.168006	0.152234	-0.345652	-0.076562
73	H	-0.026965	0.067009	-0.284429	-0.065833
74	H	-0.406171	-0.084972	-0.207039	0.013925
75	H	-0.381564	0.085598	-0.249415	0.010683

Table S6: Optimized geometry of the PIM-AMEL-TB including the ESP calculation results.

Atom		Coordinates			Charge
Number	Element	x	y	z	ESP
1	C	0.609366	-0.828821	-0.121266	-0.06968
2	C	0.570867	-0.770642	-0.241042	-0.17609
3	C	0.464615	-0.821947	-0.315355	-0.282167
4	C	0.39807	-0.936161	-0.269	0.443368
5	C	0.436243	-0.994191	-0.147769	-0.162374
6	C	0.540703	-0.940512	-0.074142	-0.169386
7	C	0.202264	1.40159	0.087528	-0.162266
8	C	0.27127	1.293377	0.140227	-0.07379
9	C	0.232333	1.235296	0.260884	-0.311961
10	C	0.122585	1.288387	0.329886	0.511443
11	C	0.053134	1.396944	0.276233	-0.279912
12	C	0.092227	1.453044	0.155749	-0.045376
13	C	0.264032	1.207335	-0.487566	-0.143276
14	C	0.337213	1.089673	-0.491047	-0.114641
15	C	0.370498	1.021824	-0.373874	-0.295364
16	C	0.331604	1.075945	-0.250714	0.515453
17	C	0.256848	1.193821	-0.247432	-0.316992
18	C	0.222726	1.258814	-0.364913	-0.068505
19	C	0.23103	0.806189	-0.129635	-0.28906
20	C	0.323352	0.75339	-0.220323	0.456721
21	C	0.256648	0.940641	-0.06426	0.603695
22	C	0.477386	0.91884	-0.146737	0.595755
23	N	0.446158	0.820891	-0.24984	-0.799158
24	N	0.367719	1.012949	-0.127215	-0.775767
25	C	0.445862	0.890193	-0.379061	0.632262
26	N	-0.103311	0.632899	0.063114	-0.539659

27	C	-0.201777	0.633949	0.153596	0.809056
28	N	-0.312337	0.559557	0.149612	-0.898876
29	C	-0.318636	0.475989	0.044527	0.893427
30	N	-0.227326	0.465058	-0.051782	-0.699491
31	C	-0.122314	0.547781	-0.037335	0.62934
32	N	-0.029876	0.539411	-0.136741	-0.476988
33	N	-0.430916	0.399005	0.043047	-0.504791
34	N	-0.191562	0.718405	0.259829	-0.515962
35	C	0.08749	0.613789	-0.15958	0.305591
36	C	-0.474761	0.296734	-0.042602	0.334996
37	C	0.114758	0.736037	-0.098926	-0.212165
38	C	0.295198	0.631215	-0.281523	-0.197149
39	C	0.178831	0.562478	-0.252405	-0.300243
40	C	-0.593857	0.232879	-0.007962	-0.343857
41	C	-0.646014	0.129048	-0.084067	-0.219856
42	C	-0.578857	0.088874	-0.200108	0.465501
43	C	-0.460416	0.153381	-0.234955	-0.245466
44	C	-0.407334	0.255582	-0.157966	-0.257818
45	C	-0.600942	-0.216036	-0.138945	-0.259335
46	C	-0.714709	-0.174197	-0.069413	0.41603
47	C	-0.558616	-0.143422	-0.265348	0.720259
48	C	-0.770866	-0.039472	-0.257292	0.564185
49	N	-0.795821	-0.065781	-0.116112	-0.792697
50	N	-0.629419	-0.016219	-0.283248	-0.838573
51	C	-0.773993	0.058977	-0.042237	0.6377
52	C	-0.083362	0.802094	0.295125	0.219798
53	C	-0.110552	0.92691	0.349961	-0.196247
54	C	-0.008263	1.011785	0.391738	-0.344926
55	C	0.124558	0.969366	0.380439	0.446599
56	C	0.151493	0.844275	0.324675	-0.266435
57	C	0.049633	0.760998	0.282126	-0.144691
58	C	0.306893	1.115772	0.317119	0.622032
59	C	0.186109	1.154531	0.516334	0.616959
60	N	0.080358	1.234138	0.455518	-0.857363
61	N	0.233145	1.050589	0.425821	-0.793109
62	C	-0.039483	1.150172	0.445933	0.74492
63	N	-0.320568	-0.535506	-0.07559	-0.688076
64	C	-0.21288	-0.611091	-0.103014	0.565402
65	N	-0.161228	-0.704958	-0.024081	-0.456707
66	C	-0.227305	-0.722541	0.090771	0.680337
67	N	-0.337019	-0.656775	0.128649	-0.853693
68	C	-0.378274	-0.563599	0.041141	0.955423
69	N	-0.489238	-0.495946	0.081695	-0.620947

70	N	-0.182132	-0.815732	0.179115	-0.451849
71	N	-0.154549	-0.585104	-0.223035	-0.431743
72	C	-0.56086	-0.389136	0.02532	0.403652
73	C	-0.067836	-0.897916	0.171644	0.272772
74	C	-0.525704	-0.323736	-0.092395	-0.308717
75	C	-0.750096	-0.240697	0.048006	-0.194535
76	C	-0.674229	-0.345889	0.09537	-0.314622
77	C	-0.072129	-1.022131	0.234216	-0.252577
78	C	0.03843	-1.106722	0.235765	-0.346603
79	C	0.155402	-1.067434	0.169862	0.434893
80	C	0.15995	-0.942272	0.10838	-0.215968
81	C	0.050816	-0.857234	0.109792	-0.191014
82	C	0.3773	-1.114437	0.255515	0.758197
83	C	0.233226	-1.291097	0.192515	0.535869
84	N	0.165009	-1.301889	0.320745	-0.861955
85	N	0.270011	-1.153166	0.163167	-0.753022
86	C	0.032584	-1.240479	0.307309	0.772157
87	C	-0.042447	-0.644394	-0.284593	0.276496
88	C	0.000626	-0.773943	-0.257498	-0.192237
89	C	0.111303	-0.82727	-0.322997	-0.340736
90	C	0.178409	-0.752015	-0.420003	0.499057
91	C	0.133575	-0.622993	-0.448266	-0.249439
92	C	0.02542	-0.569166	-0.380991	-0.261585
93	C	0.419764	-0.754297	-0.443687	0.746204
94	C	0.293869	-0.95025	-0.482632	0.711896
95	N	0.291807	-0.995356	-0.344189	-0.835126
96	N	0.290639	-0.804865	-0.491504	-0.867459
97	C	0.15878	-0.96748	-0.289251	0.701836
98	C	0.396932	-1.142479	0.635403	-0.115769
99	C	0.424742	-1.107115	0.504266	-0.059861
100	C	0.348502	-1.156448	0.398488	-0.397153
101	C	0.24379	-1.245287	0.425961	0.565053
102	C	0.215821	-1.28058	0.558076	-0.247897
103	C	0.291243	-1.229123	0.662262	-0.152011
104	H	0.691886	-0.787142	-0.064473	0.097671
105	H	0.622664	-0.682165	-0.277275	0.1022
106	H	0.383474	-1.082103	-0.112116	0.090109
107	H	0.569839	-0.987064	0.019492	0.109686
108	H	0.234074	1.445656	-0.006407	0.112312
109	H	0.35506	1.250672	0.085602	0.066318
110	H	-0.030576	1.437641	0.332068	0.133287
111	H	0.037811	1.53788	0.115526	0.087401
112	H	0.238496	1.258367	-0.579839	0.105288

113	H	0.367801	1.047871	-0.58665	0.097022
114	H	0.22711	1.233948	-0.151084	0.180207
115	H	0.16488	1.350557	-0.360969	0.096427
116	H	0.279823	0.92634	0.041973	-0.018617
117	H	0.166775	1.002502	-0.068438	-0.080948
118	H	0.49675	0.867783	-0.052082	-0.047262
119	H	0.567561	0.973097	-0.17616	-0.03603
120	H	0.549972	0.907118	-0.409276	-0.07492
121	H	0.401383	0.824601	-0.454284	-0.069956
122	H	-0.051876	0.467923	-0.204452	0.309383
123	H	-0.489825	0.418137	0.122743	0.307584
124	H	-0.274028	0.720536	0.317865	0.33737
125	H	0.044562	0.776862	-0.027624	0.107084
126	H	0.367416	0.589543	-0.350985	0.128438
127	H	0.159112	0.466927	-0.300194	0.151732
128	H	-0.646286	0.263467	0.082402	0.138114
129	H	-0.409796	0.122775	-0.325977	0.145049
130	H	-0.315439	0.304648	-0.186011	0.16276
131	H	-0.578054	-0.206347	-0.353098	-0.088626
132	H	-0.451107	-0.124177	-0.263255	-0.094872
133	H	-0.803797	-0.124943	-0.316882	-0.021966
134	H	-0.828675	0.048283	-0.287287	-0.015117
135	H	-0.86024	0.124574	-0.05944	-0.081788
136	H	-0.771727	0.036973	0.064761	-0.061058
137	H	-0.213848	0.960118	0.357549	0.108518
138	H	0.254738	0.8114	0.31825	0.152793
139	H	0.072049	0.664249	0.239412	0.122398
140	H	0.326354	1.042953	0.238082	-0.068401
141	H	0.404626	1.146936	0.355955	-0.067105
142	H	0.270368	1.218992	0.542436	-0.026084
143	H	0.147456	1.109051	0.607864	-0.030776
144	H	-0.084385	1.142714	0.545751	-0.092255
145	H	-0.112667	1.199909	0.381855	-0.089384
146	H	-0.521995	-0.528685	0.171376	0.327655
147	H	-0.243511	-0.828414	0.258173	0.313948
148	H	-0.197206	-0.507543	-0.271439	0.297784
149	H	-0.437889	-0.356329	-0.146311	0.142732
150	H	-0.839384	-0.209303	0.101099	0.129614
151	H	-0.703381	-0.39653	0.186971	0.15646
152	H	-0.163723	-1.053555	0.283833	0.121049
153	H	0.25127	-0.912161	0.057996	0.149017
154	H	0.055753	-0.761046	0.060897	0.111189
155	H	0.39136	-1.006226	0.250051	-0.099493

156	H	0.470638	-1.160497	0.220768	-0.092813
157	H	0.323911	-1.352022	0.193825	-0.021989
158	H	0.166991	-1.328965	0.1143	-0.011858
159	H	-0.032266	-1.309927	0.252474	-0.098314
160	H	-0.01104	-1.228106	0.406709	-0.098085
161	H	-0.050319	-0.832229	-0.182025	0.097367
162	H	0.184525	-0.565546	-0.524943	0.143112
163	H	-0.007576	-0.468141	-0.403447	0.147111
164	H	0.412067	-0.646385	-0.428326	-0.096224
165	H	0.494169	-0.76988	-0.522801	-0.106892
166	H	0.384581	-0.986254	-0.531801	-0.06064
167	H	0.207267	-0.992288	-0.534317	-0.060215
168	H	0.088648	-1.041576	-0.329535	-0.094057
169	H	0.160617	-0.981499	-0.180974	-0.066549
170	H	0.456947	-1.102554	0.716487	0.104352
171	H	0.506143	-1.038245	0.482978	0.06887
172	H	0.13521	-1.350742	0.577063	0.128057
173	H	0.268725	-1.257706	0.764492	0.123257

8. Recyclability test

A glass vial was charged with a mixture of benzaldehyde (25 mmol), malononitrile (25 mmol) and 10 mL of ethanol. Then, **AMEL-TB-PIM** (1 mol%) was added and the reaction mixture was stirred at room temperature for 1 h. After this period, the reaction was analysed by NMR and the catalyst was recovered from the reaction by simple filtration, refluxed in different solvents (acetone, DCM and methanol), dried in a vacuum oven (at 100 ^{0}C for 20 h) and reused. This procedure was repeated for more five extra cycles. The obtained product was separated and weighed, to be sure that the yield matched with the conversion seen by ^{1}H NMR. To ensure that the structure of the polymer has not changed during the recycling tests, we occasionally repeated the physical characterisation (BET and FT-IR), finding that the physical properties were not affected.

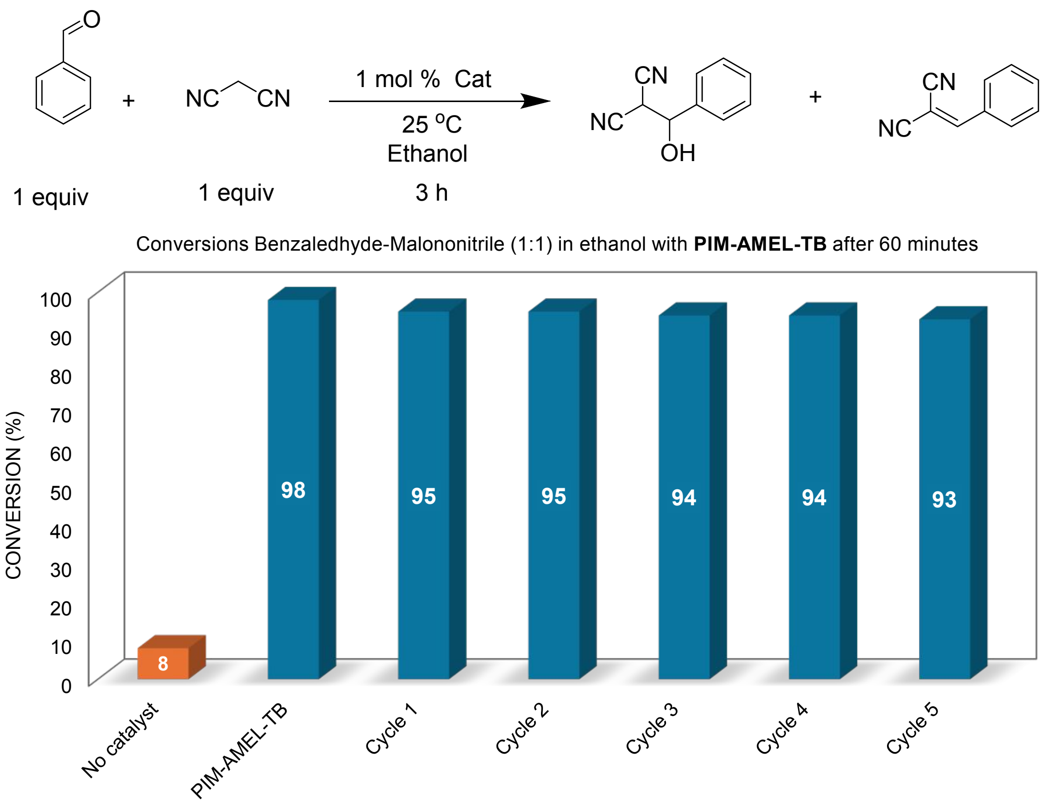

Figure S17. Recyclability test of PIM-AMEL-TB after the Knoevenagel reaction using benzaldehyde: malononitrile 1:1 and ethanol as a solvent for 60 minutes.

9. TGA curves

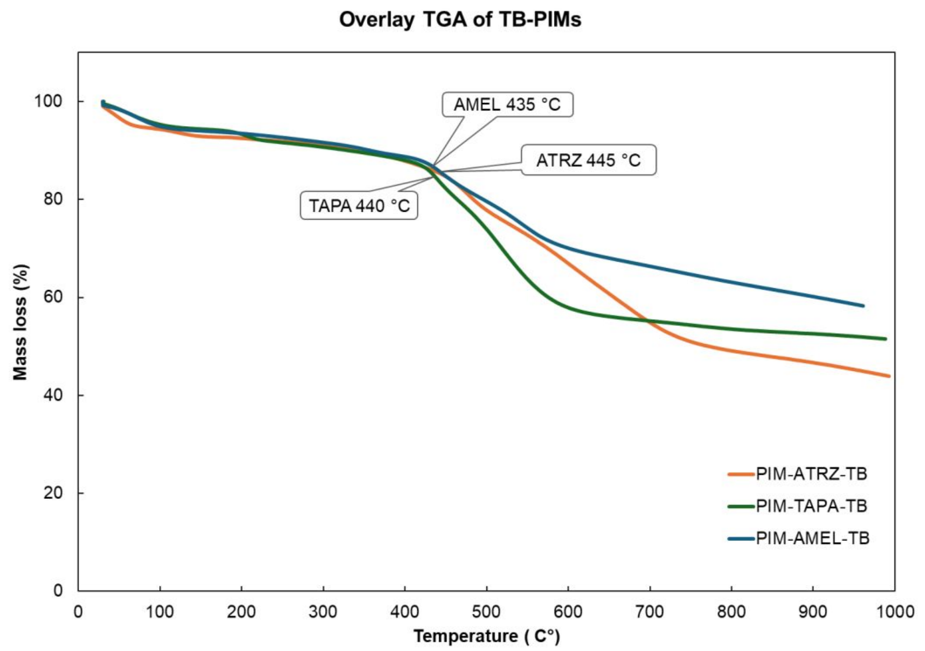

Figure S18. Overlay of the TGA of the three TB polymer PIM-ATRZ-TB, PIM-AMEL-TB and PIM-TAPA-TB. Temperature increments 10 °C min^{-1} up to 1000 °C under nitrogen flow of 20 mL min^{-1}.

10. FT-IR

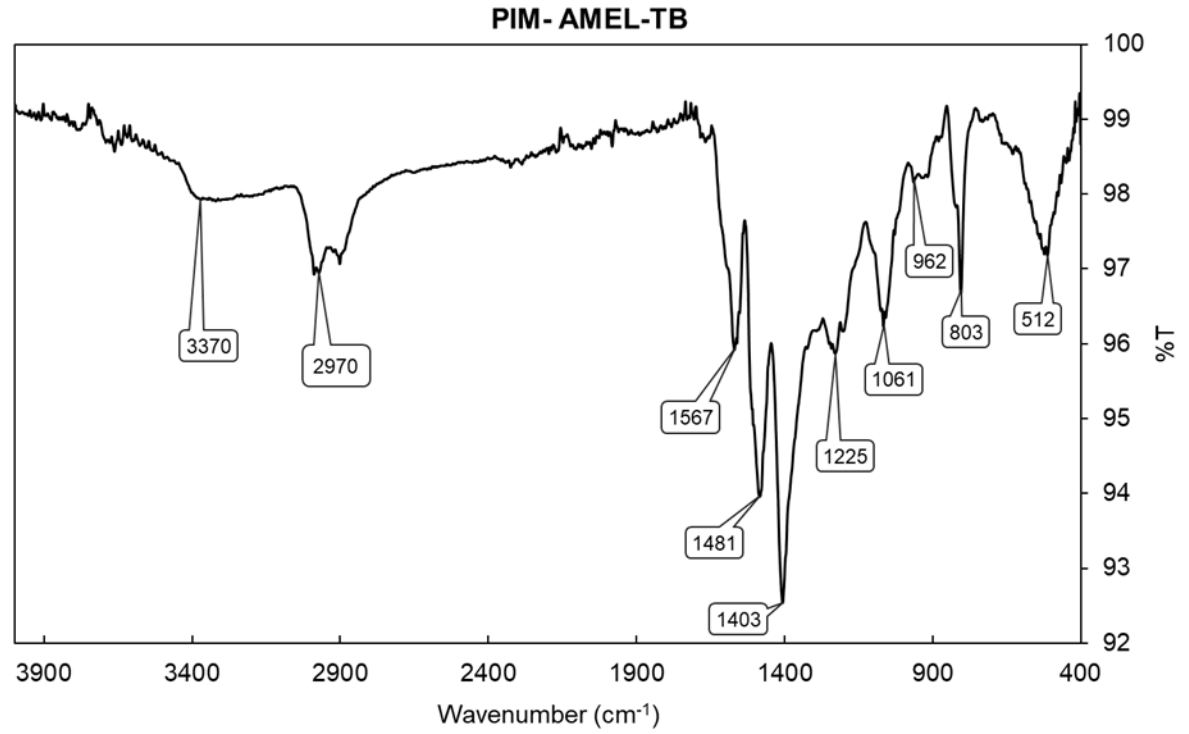

Figure S19. FT-IR trace of PIM-AMEL-TB

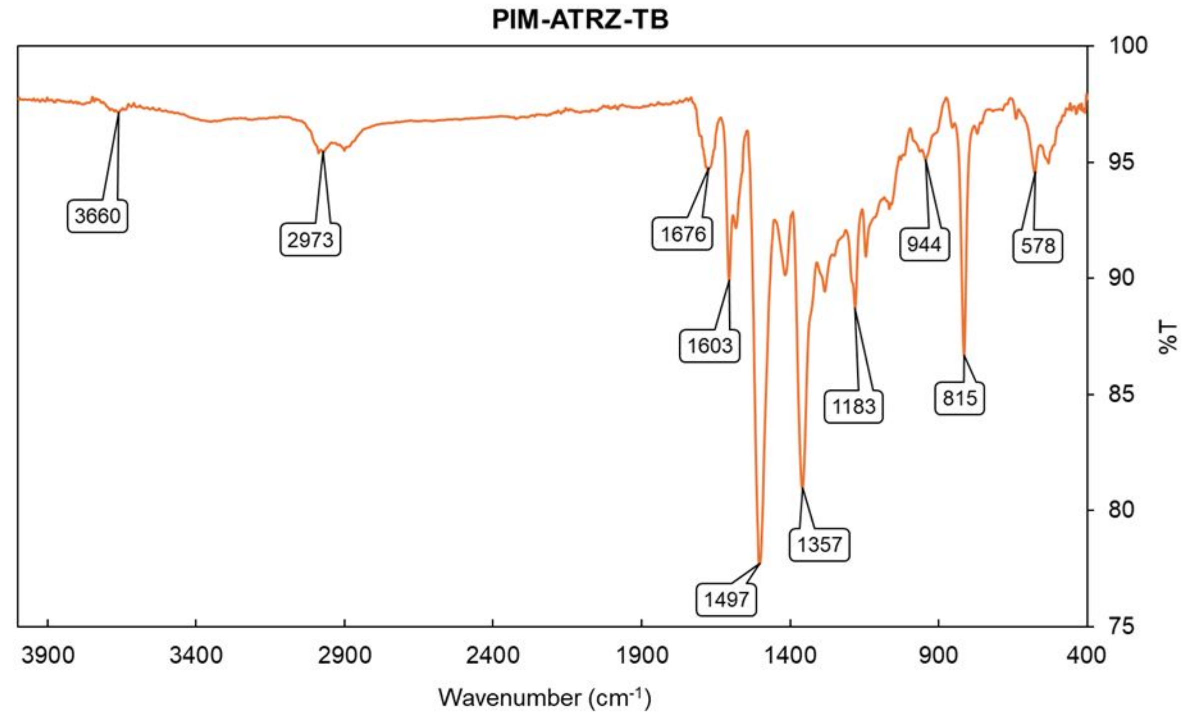

Figure S20. FT-IR trace of PIM-ATRZ-TB

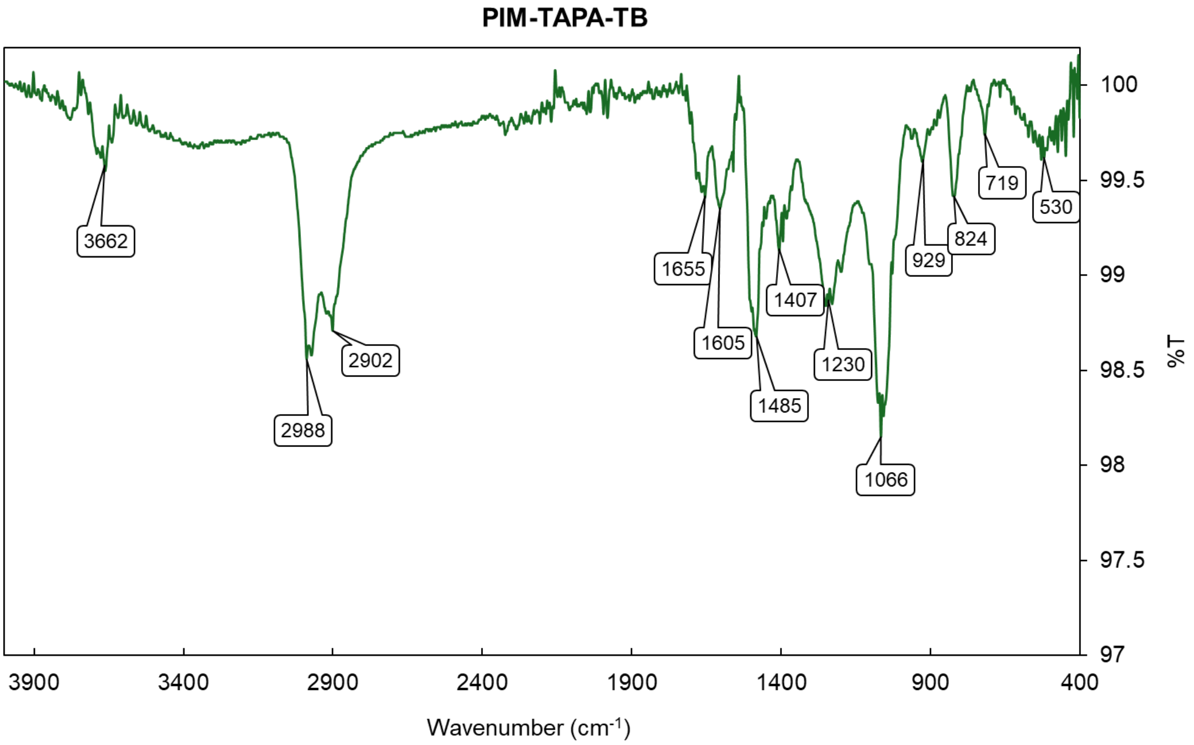

Figure S21. FT-IR trace of PIM-TAPA-TB

References

1. Y. Zhi, Z. Li, X. Feng, H. Xia, Y. Zhang, Z. Shi, Y. Mu and X. Liu, *J. Mater. Chem. A*, 2017, **5**, 22933-22938.
2. J. Feng, B. Liang, D. Wang, H. Wu, L. Xue and X. Li, *Langmuir*, 2008, **24**, 11209-11215.
3. C. López-Lira, R. A. Tapia, A. Herrera, M. Lapier, J. D. Maya, J. Soto-Delgado, A. G. Oliver, A. G. Lappin and E. Uriarte, *Bioorg. Chem.*, 2021, **111**, 104823.
4. P. Sun, Y. Chen, B. Sun, H. Zhang, K. Chen, H. Miao, Q. Fan and W. Huang, *ACS Appl. Bio Mater.*, 2021, **4**, 4542-4548.
5. L. Trupp, A. C. Bruttomesso and B. C. Barja, *New J. Chem.*, 2020, **44**, 10973-10981.
6. G. Gattuso, G. Grasso, N. Marino, A. Notti, A. Pappalardo, S. Pappalardo and M. F. Parisi, *Journal*, 2011.
7. M. Carta, M. Croad, K. Bugler, K. J. Msayib and N. B. McKeown, *Polymer Chemistry*, 2014, **5**, 5262-5266.
8. A. R. Antonangelo, N. Hawkins, E. Tocci, C. Muzzi, A. Fuoco and M. Carta, *Journal of the American Chemical Society*, 2022, **144**, 15581-15594.
9. E. Aprà, E. J. Bylaska, W. A. de Jong, N. Govind, K. Kowalski, T. P. Straatsma, M. Valiev, H. J. J. van Dam, Y. Alexeev, J. Anchell, V. Anisimov, F. W. Aquino, R. Atta-Fynn, J. Autschbach, N. P. Bauman, J. C. Becca, D. E. Bernholdt, K. Bhaskaran-Nair, S. Bogatko, ... and R. J. Harrison, *J. Chem. Phys.*, 2020, **152**.
10. T. Yanai, D. P. Tew and N. C. Handy, *Chem. Phys. Lett.*, 2004, **393**, 51-57.
